# Uncovering novel mutational signatures by *de novo* extraction with SigProfilerExtractor

**DOI:** 10.1016/j.xgen.2022.100179

**Published:** 2022-11-09

**Authors:** S.M. Ashiqul Islam, Marcos Díaz-Gay, Yang Wu, Mark Barnes, Raviteja Vangara, Erik N. Bergstrom, Yudou He, Mike Vella, Jingwei Wang, Jon W. Teague, Peter Clapham, Sarah Moody, Sergey Senkin, Yun Rose Li, Laura Riva, Tongwu Zhang, Andreas J. Gruber, Christopher D. Steele, Burçak Otlu, Azhar Khandekar, Ammal Abbasi, Laura Humphreys, Natalia Syulyukina, Samuel W. Brady, Boian S. Alexandrov, Nischalan Pillay, Jinghui Zhang, David J. Adams, Iñigo Martincorena, David C. Wedge, Maria Teresa Landi, Paul Brennan, Michael R. Stratton, Steven G. Rozen, Ludmil B. Alexandrov

**Affiliations:** 1 Department of Cellular and Molecular Medicine, UC San Diego, La Jolla, CA 92093, USA; 2 Department of Bioengineering, UC San Diego, La Jolla, CA 92093, USA; 3 Moores Cancer Center, UC San Diego, La Jolla, CA 92037, USA; 4 Centre for Computational Biology and Programme in Cancer & Stem Cell Biology, Duke NUS Medical School, Singapore 169857, Singapore; 5 NVIDIA Corporation, 2788 San Tomas Expressway, Santa Clara, CA 95051, USA; 6 Cancer, Ageing and Somatic Mutation, Wellcome Sanger Institute, Wellcome Genome Campus, Cambridge CB10 1SA, UK; 7 Genetic Epidemiology Group, International Agency for Research on Cancer, Cedex 08, 69372 Lyon, France; 8 Departments of Radiation Oncology and Cancer Genetics, City of Hope Comprehensive Cancer Center, Duarte, CA, USA; 9 Division of Cancer Epidemiology and Genetics, National Cancer Institute, Bethesda, MD 20892, USA; 10 Big Data Institute, Nuffield Department of Medicine, University of Oxford, Oxford OX3 7LF, UK; 11 Manchester Cancer Research Centre, The University of Manchester, Manchester M20 4GJ, UK; 12 Research Department of Pathology, Cancer Institute, University College London, London WC1E 6BT, UK; 13 Department of Computational Biology, St. Jude Children’s Research Hospital, Memphis, TN 38105, USA; 14 Theoretical Division, Los Alamos National Laboratory, Los Alamos, NM 87545, USA; 15 Department of Cellular and Molecular Pathology, Royal National Orthopaedic Hospital NHS Trust, Stanmore, Middlesex HA7 4LP, UK; 16 Department of Biology, University of Konstanz, Universitaetsstrasse 10, D-78464 Konstanz, Germany

**Keywords:** mutagenesis, mutational signatures, cancer genomics, genomics

## Abstract

Mutational signature analysis is commonly performed in cancer genomic studies. Here, we present SigProfilerExtractor, an automated tool for *de novo* extraction of mutational signatures, and benchmark it against another 13 bioinformatics tools by using 34 scenarios encompassing 2,500 simulated signatures found in 60,000 synthetic genomes and 20,000 synthetic exomes. For simulations with 5% noise, reflecting high-quality datasets, SigProfilerExtractor outperforms other approaches by elucidating between 20% and 50% more true-positive signatures while yielding 5-fold less false-positive signatures. Applying SigProfilerExtractor to 4,643 whole-genome- and 19,184 whole-exome-sequenced cancers reveals four novel signatures. Two of the signatures are confirmed in independent cohorts, and one of these signatures is associated with tobacco smoking. In summary, this report provides a reference tool for analysis of mutational signatures, a comprehensive benchmarking of bioinformatics tools for extracting signatures, and several novel mutational signatures, including one putatively attributed to direct tobacco smoking mutagenesis in bladder tissues.

## Highlights

• Most advanced bioinformatics tool for *de novo* extraction of mutational signatures
• Comprehensive benchmarking of 14 *de novo* extraction tools with and without noise
• Analysis of 23,827 sequenced cancers revealing four novel mutational signatures
• Novel signature attributed to direct tobacco smoking mutagenesis in bladder tissues

## Graphical abstract

**Figure:**
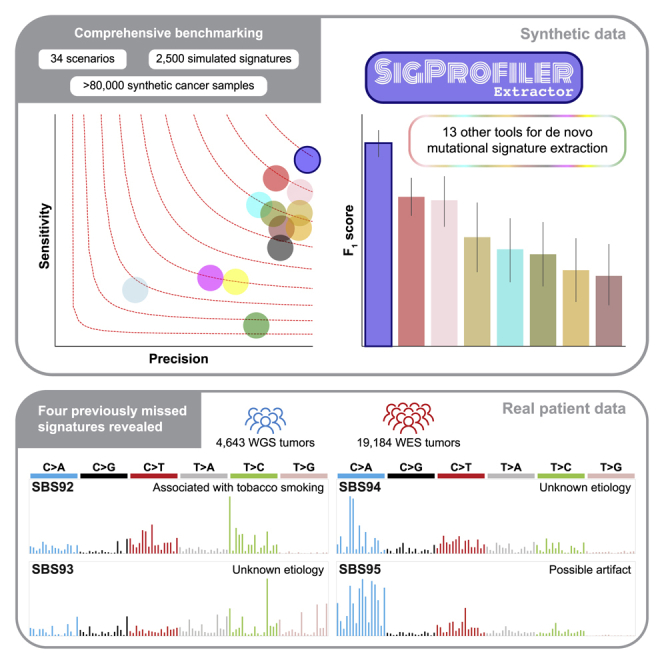

## Teaser

Islam et al. present SigProfilerExtractor, a novel computational tool for *de novo* extraction of mutational signatures. Using more than 80,000 synthetic cancer samples, the authors demonstrate that SigProfilerExtractor outperforms 13 existing approaches. Applying SigProfilerExtractor to more than 23,000 sequenced human cancers reveals novel insights about the processes leading to somatic mutation accumulation and tumorigenesis, including a novel mutational signature related to tobacco smoking in bladder cancer.

## Introduction

The somatic mutations found in a cancer genome are the cumulative result of all endogenous and exogenous mutational processes that have been operative through the lineage of a cancer cell.^1^ By examining the types of mutations in *TP53* across cancers, early studies demonstrated that specific environmental carcinogens exhibit characteristic patterns of somatic mutations.^2^ The explosion of next-generation sequencing data from cancer genomes^3^ and the development of novel computational approaches^4^ have allowed separating the signatures of individual mutagenic processes operative in cancer. Large-scale analyses of cancer genomes have revealed more than 100 distinct signatures, with some attributed to exposures to environmental carcinogens, failure of DNA-repair pathways, infidelity/deficiency of replicating polymerases, iatrogenic events, and others.^5^^,^^6^^,^^7^^,^^8^^,^^9^^,^^10^^,^^11^^,^^12^ Moreover, mutational signatures have been utilized for both cancer prevention and cancer treatment.^13^^,^^14^

*De novo* extraction of mutational signatures^4^ is an unsupervised machine-learning approach where a matrix, *M*, which corresponds to the somatic mutations in a set of cancer samples under a mutational classification,^15^ is approximated by the product of two low-rank matrices, *S* and *A*. The matrix *S* reflects the set of mutational signatures, while the matrix *A* encompasses the activities of the signatures; an activity corresponds to the number of mutations contributed by a signature in a cancer sample. Algorithmically, *de novo* extraction of mutational signatures has relied on nonnegative matrix factorization (NMF)^16^ or on approaches mathematically analogous to NMF.^17^^,^^18^^,^^19^ The main advantage of NMF over other factorization approaches is its ability to yield nonnegative factors that are part of the original data, thus allowing biological interpretation of the identified nonnegative factors.^16^

Since we introduced the mathematical concept of mutational signatures,^4^ multiple computational frameworks were developed for *de novo* extraction of mutational signatures (Table 1).^12^^,^^20^^,^^22^^,^^24^^,^^25^^,^^27^^,^^28^^,^^31^^,^^32^^,^^34^^,^^35^^,^^36^^,^^38^^,^^40^ Notably, the majority of existing tools (1) predominately support the simplest mutational classification, viz., SBS-96, which encompasses single base substitutions with their immediate 5′ and 3′ sequence context;^15^ (2) lack automatic selection for the number of signatures; (3) do not identify a robust solution; (4) require pre-selection of a large number of hyperparameters; and (5) do not decompose *de novo* signatures to the set of more than 100 reference signatures available at the Catalog of Somatic Mutations in Cancer (COSMIC) database.^12^^,^^42^ Importantly, there has been no extensive benchmark of the existing tools for *de novo* extraction leading to uncertainty regarding their performance.

**Table 1:** Overview of bioinformatics tools for *de novo* extraction of mutational signatures

Tool name	Input	Platform	Factorization method	Factorization engine	GPU	Manual selection	Automatic selection	Automatic algorithm	Mutational catalog support	Plotting support	COSMIC comparison
EMu^20^	matrix	C++	EM	original implementation^20^	no	yes	yes^a^	BIC^21^	SBS-96	no	no
Maftools^22^	matrix, MAF	R-Bioconductor	NMF	NMF R package^23^	no	yes	no	–	SBS-96	SBS-96	1 to 1
MutationalPatterns^24^	matrix, VCF	R-Bioconductor	NMF	NMF R package^23^	no	yes	no	–	SBS-96, SBS-192	SBS-96, SBS-192	1 to 1
MutSignatures^25^	matrix, VCF, MAF	R	NMF	Brunet et al.^26^	no	no	no	–	SBS-96	SBS-96	1 to 1
MutSpec^27^	matrix, VCF, custom	Galaxy, Perl, R	NMF	NMF R package^23^	no	yes	no	–	SBS-96, SBS-192	SBS-96, SBS-192	1 to 1
SigFit^28^	matrix	R	Bayesian inference	Stan R package^29^	no	yes	yes^a^	Elbow method^30^	SBS-96	SBS-96, SBS-192	1 to 1
SigMiner^31^	matrix, MAF	R	(automatic) Bayesian NMF, (manual) NMF	(automatic) SignatureAnalyzer implementation,^32^ (manual) NMF R package^23^	no	yes^a^	yes	ARD^33^	SBS-96, DBS-78, ID-83	generic	1 to 1
SignatureAnalyzer^32^^,^^34^	matrix, MAF	R (CPU),^18^ Python (GPU)^19^	Bayesian NMF	original implementation^32^^,^^34^	yes	no	yes	ARD^33^	SBS-96, DBS-78, ID-83	SBS-96, DBS-78, ID-83	1 to 1
SignatureToolsLib^35^	matrix, VCF, custom	R	NMF	NMF R package^23^	no	yes	no	–	SBS-96, DBS-78, ID-83, SV-32	SBS-96, SV-32, generic	1 to 2
SigneR^36^	matrix, VCF	R-Bioconductor, C++	Bayesian NMF	original implementation^36^	no	yes	yes^a^	BIC^21^	SBS-96	SBS-96	no
SigProfilerExtractor	matrix, VCF, MAF, custom	Python, R wrapper	NMF	(current work) original implementation	yes	yes	yes^a^	NMFk^37^	SBS-96, DBS-78, ID-83, CN-48, others,^15^ any	SBS-96, DBS-78, ID-83, CN-48, SV-32, others,^15^ generic	1 to many
SigProfiler_PCAWG^12^	matrix, VCF, MAF, custom	Python, MATLAB	NMF	Brunet et al.^26^	no	yes	no	–	SBS-96, DBS-78, ID-83, others,^15^ any	SBS-96, DBS-78, ID-83	no
SomaticSignatures^38^	matrix, VCF	R-Bioconductor	NMF, PCA	NMF R package^23^ pcaMethods R package^39^	no	yes	no	–	SBS-96	SBS-96	no
TensorSignatures^40^	VCF	Python	NTF	TensorFlow^41^	yes	yes	yes^a^	BIC^21^	tensor	SBS-96 with strand bias	no

Tools are ordered alphabetically. 1 to 1 refers to one *de novo* signature being matched with exactly one COSMIC signature; 1 to 2 refers to one *de novo* signature being matched with a combination of up to two COSMIC signatures; 1 to many refers to one *de novo* signature being matched with a combination of one or more COSMIC signatures. MAF, mutation annotation format; VCF, variant call format; EM, expectation maximization algorithm; NMF, nonnegative matrix factorization; PCA, principal component analysis; NTF, nonnegative tensor factorization; ARD, automatic relevance determination; BIC, Bayesian information criterion; COSMIC, catalog of somatic mutations in cancer; SBS, single base substitutions; DBS, doublet base substitutions; ID, small insertions and deletions; CN, copy number; SV, structural variants.

a The default approach for selecting the total number of signatures when a tool supports both manual and automatic selection.

To address these limitations, here we present SigProfilerExtractor—a reference tool for *de novo* extraction of mutational signatures. SigProfilerExtractor allows analysis of all types of mutational classifications, performs automatic selection of the number of signatures, yields robust solutions, requires only minimum setup, and decomposes *de novo* extracted signatures to known COSMIC signatures. A comprehensive benchmark including 3,608 unique matrix decompositions with SigProfilerExtractor and 13 other tools across a total of 34 distinct scenarios reveals that SigProfilerExtractor is robust to noise and that it outperforms all other computational tools for *de novo* extraction of mutational signatures (Tables S1, S2, S3, S4, and S5). Applying SigProfilerExtractor to the recently published set of 2,778 whole-genome-sequenced (WGS) cancers from the Pan-Cancer Analysis of Whole Genomes (PCAWG) project^43^ and an additional curated collection of 1,865 WGS and 19,184 whole-exome-sequenced (WES) cancers (Table S8) elucidates four novel mutational signatures. Two of the signatures are confirmed in independent cohorts, and a putative etiology of tobacco-associated mutagenesis is attributed to one of these signatures (SBS92).

## Results

### Overview of SigProfilerExtractor

**Figure 1:**
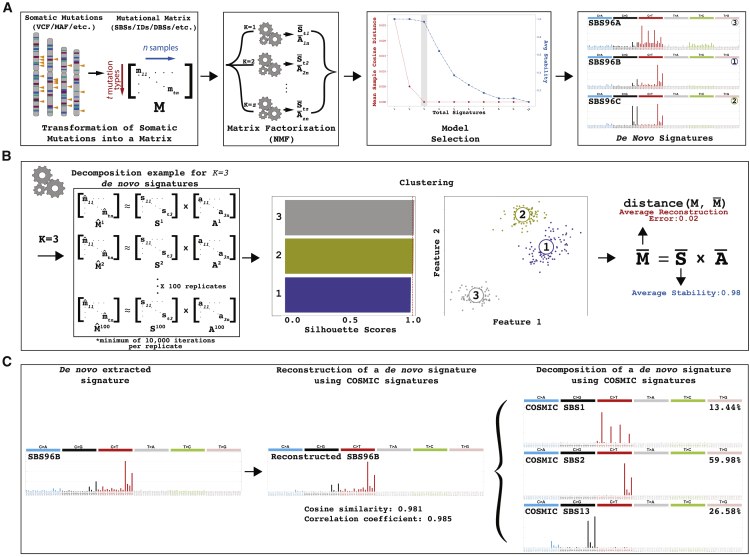
Overview of SigProfilerExtractor (A) SigProfilerExtractor’s general workflow is outlined starting from an input of somatic mutations and resulting in an output of *de novo* mutational signatures. An example is shown for a solution with three *de novo* signatures. Somatic mutations are first converted into a mutational matrix *M*. Subsequently, the matrix is factorized with different ranks using nonnegative matrix factorization. Model selection is applied to identify the optimal factorization rank based on each solution’s stability and its reconstruction of the original data. (B) Schematic representation for an example decomposition with a factorization rank of *k = 3* reflecting three operative mutational signatures. By default, SigProfilerExtractor performs 100 independent nonnegative matrix factorizations with the matrix *M* being Poisson resampled and normalized (denoted by “ˆ”) prior to each factorization. Partition clustering of the 100 factorizations is used to evaluate the factorization stability rank, measured in silhouette values; clustering can also be presented as two-dimensional projections revealing more similar mutational signatures as shown for the three example signatures. The centroid of the clustered solutions (denoted by “–”) is compared with the original matrix *M*. (C) All identified *de novo* signatures are matched to a combination of known COSMIC mutational signatures. An example is given for *de novo* extracted signature SBS96B, which matches a combination of COSMIC signatures SBS1, SBS2, and SBS13.

SigProfilerExtractor is implemented as a Python package, with an R wrapper, allowing users to run it in both Python and R environments (STAR Methods). By default, the tool requires only a single parameter—the input dataset containing the mutational catalogs of interest. SigProfilerExtractor supports most used formats outputted by variant-calling algorithms, which are internally converted^15^ into a matrix, *M*. By default, the tool decomposes the matrix *M* searching for an optimal solution for the number of operative signatures, *k*, between 1 and 25 mutational signatures (Figure 1A). For each decomposition, SigProfilerExtractor performs 100 independent factorizations and, for each repetition, the matrix *M* is first Poisson resampled and normalized and, subsequently, factorized with the multiplicative update NMF algorithm^16^ by minimizing an objective function based on the Kullback-Leibler divergence measure^44^ (Figure 1B). Custom partition clustering, which utilizes the Hungarian algorithm^45^ for comparing different repetitions, is applied to the 100 factorizations to identify stable solutions.^46^ Specifically, the centroids of stable clusters are selected as optimal solutions, thus making these solutions resistant to fluctuations in the input data and the lack of uniqueness of NMF.^47^ Lastly, when applicable, the optimal set of *de novo* signatures are matched to the set of reference COSMIC signatures (Figure 1C), with any *de novo* signature reported as novel when it cannot be decomposed by a combination of known COSMIC signatures.

### Framework for benchmarking tools for *de novo* extraction

To benchmark tools for *de novo* extraction of mutational signatures, more than 60,000 unique synthetic cancer genomes and 20,000 cancer exomes were generated with known ground-truth mutational signatures (STAR Methods). These synthetic data included 32 noiseless scenarios and two scenarios with different levels of noise. Each scenario contained between 3 and 39 known signatures operative in 200 to 2,700 simulated cancer genomes (Tables S1, S2, S3, S4, and S5). Some scenarios were generated up to 20 times to account for variability in the simulations. While most noiseless scenarios (20/32) were based on SBS-96 mutational classification, we also generated 12 scenarios using extended classifications, i.e., matrices with more than 96 mutational channels (Table S2). To avoid bias in evaluating each tool’s performance, three sets of SBS-96 signatures were used in generating the synthetic data: (1) COSMICv3 reference signatures,^12^ (2) signatures previously extracted by SignatureAnalyzer (SA),^12^ and (3) randomly generated signatures. Most of the noiseless scenarios were designed to mimic the activities of mutational signatures in specific cancer types, with four scenarios emulating a single cancer type, 16 scenarios a combination of two cancer types, and two scenarios mimicking the analysis of a pan-cancer dataset. In addition, randomly generated signatures displaying different distributions and exposures were used in 10 noiseless scenarios and in the noise scenarios, which were generated up to 20 times. Some of the scenarios included combinations of signatures that represent a challenge for *de novo* extraction, including mutational signatures with overlapping profiles in specific contexts or exhibiting flat featureless profiles. For presentation simplicity, scenarios were labeled based on their complexity as easy, medium, or hard. Easy scenarios were generated using ≤5 signatures and provide a good indication of each tool’s performance on approximately 7.4% of human cancer types (e.g., brain tumors). Medium scenarios contained 11 to 21 signatures and biologically reflect 15.9% of cancer types (e.g., cervical cancer). Hard scenarios have ≥25 signatures and reflect 59.5% of human cancer types (e.g., breast cancer) as well as pan-cancer datasets. In addition to the 32 noiseless scenarios, one whole-genome SBS-96 scenario with five different levels of noise, ranging between 0% and 10%, was included in the benchmark (STAR Methods). Further, an SBS-96-based whole-exome scenario with 5% noise was also included.

To compare the performance between different tools, we developed a standard set of evaluation metrics (Figure S1). Specifically, each *de novo* extracted signature is classified as either a true positive (TP), false positive (FP), or false negative (FN) signature. An extracted signature is considered TP if it matches one of the ground-truth signatures above a cosine similarity threshold of 0.90. In contrast, a signature is classified as FP when it has a maximum cosine similarity below 0.90 with all ground-truth signatures. Lastly, FN signatures are ground-truth signatures that were not detected in the data. These standard metrics allow calculating each tool’s precision, sensitivity, and F_1_ score. Precision is defined as $\frac{TP}{TP+FP}$, sensitivity as $\frac{TP}{TP+FN}$, and F_1_ score corresponds to a combined metric, defined as the harmonic mean of the precision and sensitivity: $2*\frac{Precision*Sensitivity}{Precision+Sensitivity}$

### Benchmarking using SBS-96 noiseless WGS data

**Figure 2:**
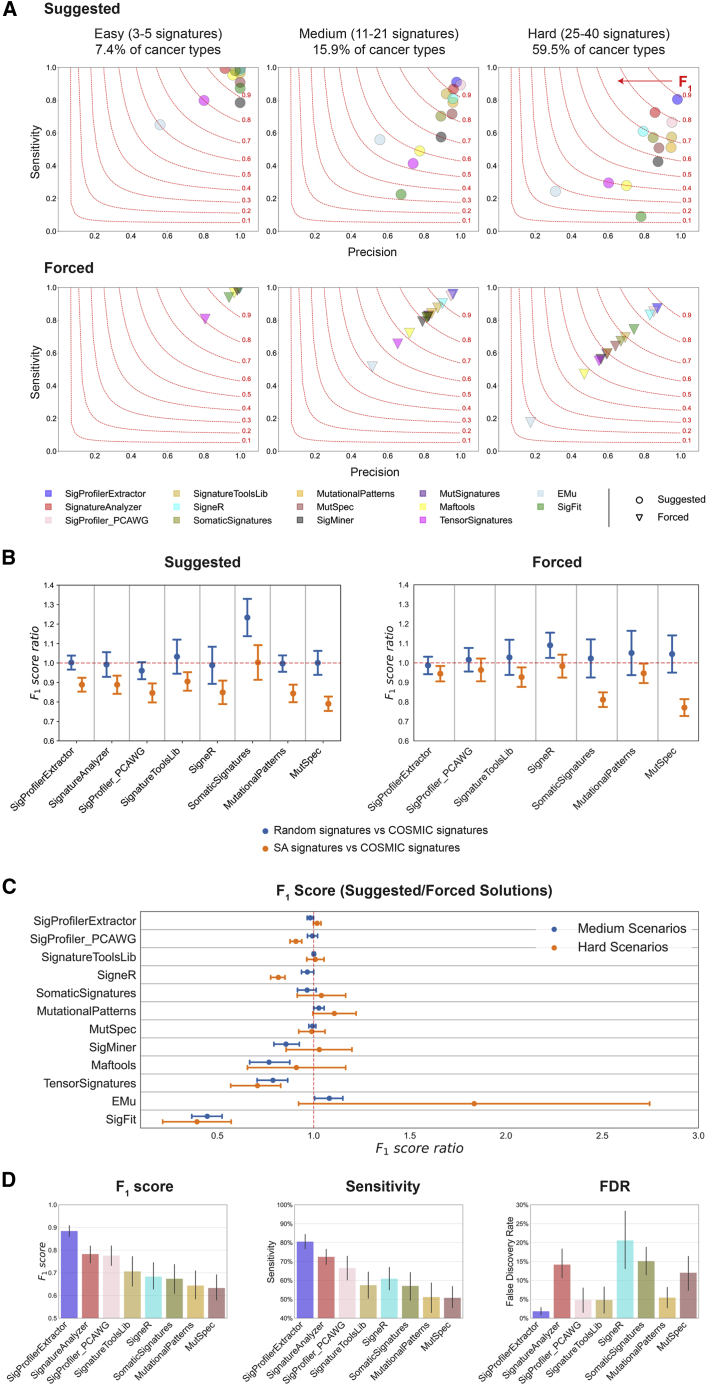
Benchmarking of bioinformatics tools for *de novo* extraction of mutational signatures using SBS-96 noiseless scenarios (A) Average precision (x axes), sensitivities (y axes), and F_1_ scores (harmonic mean of precision and sensitivity; red curves) are shown across the three types of scenarios. Different tools are displayed using circles and triangles with different colors. Circles are used to display results for suggested model selection, which most closely matches analysis of a real dataset. Triangles are used to display results for forced model selection, where tools were required to extract the known total number of ground-truth mutational signatures. All triangles are located on the diagonal, as the forced model selection results in equal numbers of false-positive and false-negative signatures. (B) Evaluating the effect of ground-truth signatures on the *de novo* extraction by different tools (x axes). Ratio of F_1_ scores (y axes) with standard errors of the mean were calculated for medium complexity scenarios simulated using COSMIC, SA, or random signatures. Ratio of approximately 1.00 indicates a similar performance between different types of signatures. (C) Evaluating the performance of *de novo* extraction between suggested and forced selection for different tools (x axes). Ratio of F_1_ scores (y axes) with standard errors of the mean was calculated for all medium and hard scenarios. Ratio of approximately 1.00 indicates a similar performance between suggested and forced model selection. (D) Summary of the performance for the top eight tools on hard SBS-96 noiseless scenarios with suggested model selection. Vertical axes reflect F_1_ score (left plot), sensitivity (middle plot), and false discovery rate (right plot), respectively. Error bars correspond to standard errors of the mean. Results from SignatureAnalyzer and MutSignatures are not displayed in (A)–(C) for forced and suggested model selections, respectively, as the tools do not support these types of analyses.

SigProfilerExtractor and 13 other tools (Table 1) were first applied to all noiseless WGS scenarios based on the SBS-96 mutational classification. The 13 tools include SignatureAnalyzer (SA) and SigProfiler_PCAWG, a legacy MATLAB/Python version of SigProfilerExtractor, which were jointly used in the PCAWG analysis of mutational signatures and the derivation of the COSMICv3 set of reference signatures.^12^ Except for MutSignatures, which can only decompose a matrix for a fixed number of signatures, all other tools were applied to each scenario by using their suggested methods for selecting the number of operative signatures. Apart from SA, which lacks this capability, all tools were also forced to extract the known number of ground-truth signatures. Results from the suggested approach reflect the expected outcome from running a tool on an unknown dataset, while results from the forced approach allow understanding limitations in each tool’s implementation. Our evaluation reveals that most tools can successfully extract mutational signatures from easy scenarios with the majority of F_1_ scores >0.90 (Figure 2A). This is perhaps unsurprising, as many of these tools used synthetic data with ≤5 signatures to evaluate their performance.^20^^,^^22^^,^^24^^,^^27^^,^^28^^,^^31^^,^^32^^,^^34^^,^^35^^,^^36^^,^^38^ In contrast, medium scenarios have proven to be a challenge for most tools with only SigProfilerExtractor, SigProfiler_PCAWG, and SA exhibiting F_1_ scores >0.90. All tools had worst performance for the hard scenarios with F_1_ scores below 0.80; only SigProfilerExtractor had an F_1_ score of ∼0.90 (Figure 2A).

To evaluate whether the type of ground-truth signatures affects the *de novo* extraction, we compared the ratio of F_1_ scores (rF_1_) from scenarios generated using COSMIC, SA, or random signatures (Figure 2B). Most tools had similar performance (rF_1_ ≈ 1) between COSMIC and random signatures and worst performance with SA signatures (rF_1_ < 1). SomaticSignatures was an exception, as it performed well on random signatures but had similarly suboptimal performance on COSMIC and SA signatures. SigProfilerExtractor outperformed all other tools regardless of whether the synthetic data were generated using COSMIC, SA, or random signatures (Table S1).

To examine the performance of *de novo* extraction between the suggested and forced selection of the total number of signatures, we evaluated rF_1_ across all medium and hard scenarios (Figure 2C). SigProfilerExtractor exhibited almost identical F_1_ scores in suggested and forced selection, indicating a good performance of the automatic selection algorithm. Most other tools had similar F_1_ scores between the suggested and forced selection, albeit with more variability across the different scenarios (Figure 2C). For example, MutSpec, one of the multiple tools based on NMF factorization, had rF_1_ ≈ 1 in both medium and hard scenarios, indicating that MutSpec is performing worse than SigProfilerExtractor (Figure 2A) not because of its algorithm for selecting the total number of signatures but likely due to its factorization approach. Other tools obtained lower F_1_ scores for suggested solutions compared with forced solutions (rF_1_ < 1), including SigneR and SigProfiler_PCAWG in the case of hard scenarios, SigMiner and Maftools for medium scenarios, and TensorSignatures and SigFit for both medium and hard scenarios. Lower F_1_ scores for suggested solutions indicate that the different approaches used by these tools for selecting the number of signatures are not optimally performing (Figure 2C). Surprisingly, EMu, the only tool based on the expectation maximization algorithm,^20^ had higher F_1_ scores for automatic solutions in some hard scenarios. Considering the overall performance of EMu (Figure 2A), this outcome likely reflects the lack of convergence during the minimization of the EMu objective function for some hard scenarios.

Overall, across all suggested extractions from noiseless WGS hard scenarios reflecting ∼60% of human cancer types, SigProfilerExtractor outperformed all other tools. SigProfilerExtractor was able to identify between 10% and 37% more TP signatures while yielding between 2.7- and 16-fold less FP signatures compared with the next seven best-performing tools (Figure 2D; Table S1).

### Extended benchmarking of the top-performing tools

**Figure 3:**
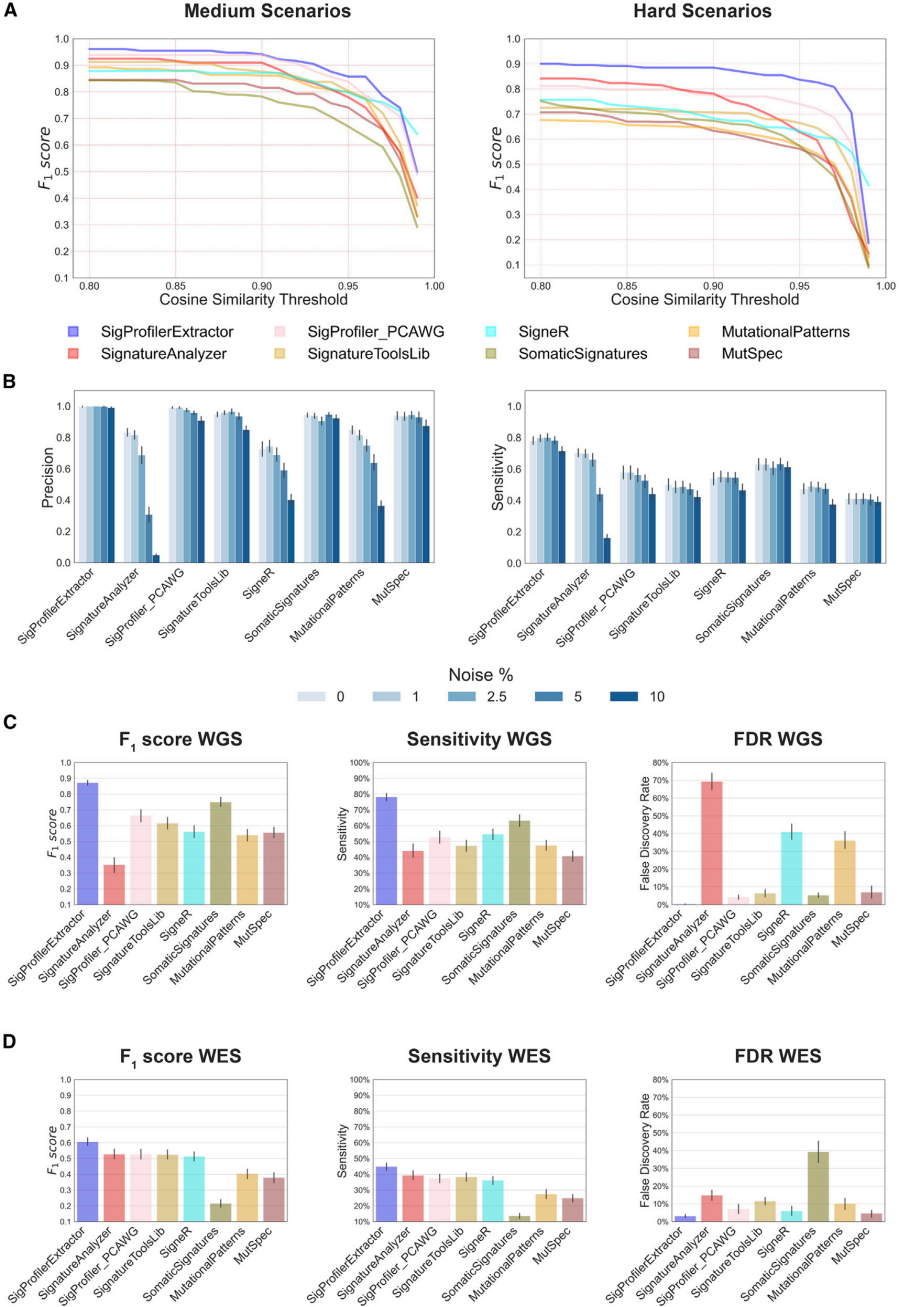
Additional evaluations of the top eight bioinformatics tools for *de novo* extraction of mutational signatures (A) Average F_1_ scores for the top eight tools based on different thresholds for cosine similarity in suggested medium and hard scenarios; thresholds for cosine similarity are used for determining true-positive signatures (Figure S1). Horizontal axes reflect the cosine similarity thresholds, while vertical axes correspond to the average F_1_ scores corresponding to cosine similarity thresholds. (B) Precision and sensitivity of the top eight tools for SBS-96 WGS scenarios with different levels of noise. Noise levels reflect the average number of somatic mutations in a cancer genome affected by additive white Gaussian noise; for example, 1% noise corresponds to approximately 1% of mutations in a sample being due to noise. Error bars correspond to standard errors of the mean. (C and D) Summary of the performance of the top eight tools on SBS-96 (C) WGS and (D) WES scenarios with 5% noise. Vertical axes reflect F_1_ score (left plot), sensitivity (middle plot), and false discovery rate (right plot), respectively. Error bars correspond to standard errors of the mean.

The reported comparisons for SBS-96 scenarios rely on a cosine similarity ≥0.90 for determining TP signatures and <0.90 for determining FP signatures. Note that a cosine similarity ≥0.90 is highly unlikely to happen purely by chance (p = 5.90 × 10^−9^), as two random nonnegative vectors are expected to have an average cosine similarity of 0.75 purely by chance.^48^ Importantly, SigProfilerExtractor’s performance does not depend on the specific value of the cosine similarity threshold (Figure 3A), as the tool consistently outperforms other approaches for TP thresholds above 0.80 (p = 0.057). Cosine similarity thresholds below 0.80 were not explored, as extracted signatures may be similar purely by chance.

Additional benchmarking was performed by generating 12 scenarios simulated using between 3 and 30 signatures with an extended number of mutational channels (STAR Methods). SigProfilerExtractor and SA are the only two tools that support analysis of custom-size matrices and provide GPU support (Table 1), thus allowing analysis of data with an extended number of mutational channels within a reasonable time frame. In contrast, all other matrix factorization tools rely solely on CPU implementations, with full runs expected to take many months for each tool applied to these scenarios. Overall, SigProfilerExtractor outperformed SA with average F_1_ scores of 0.92 and 0.85, respectively (Table S2).

To further compare SigProfilerExtractor with the other seven top-performing tools, we applied each tool to a dataset with 30 ground-truth SBS-96 signatures operative in 1,000 genomes and random noise between 0% and 10%. Analysis for each noise level was repeated 20 times to account for variability in the noise generation. SigProfilerExtractor, SomaticSignatures, MutSpec, and SignatureToolsLib were robust to noise, with mostly unaffected performance (Figure 3B; Table S3). In contrast, SigProfiler_PCAWG, SA, SigneR, and MutationalPatterns were susceptible to noise (Figure 3B). For example, 2.5% noise reduced SA’s F_1_ from 0.76 to 0.66, while 10% noise reduced its F_1_ to 0.07. Similarly, 10% noise reduced the F_1_ of SigProfiler_PCAWG from 0.71 to 0.58, the F_1_ of SigneR from 0.61 to 0.43, and the F_1_ of MutationalPatterns from 0.60 to 0.37. SA’s reduced performance on data with noise is due to its automated approach for selecting total number of signatures. SA uses automatic relevance determination (ARD)^33^ for selecting the number of signatures, with this number increasing from 26 (no noise; 30 ground-truth signatures) to 96 signatures (10% noise; Table S3). In contrast, SigProfiler_PCAWG, SigneR, and MutationalPatterns exhibit similar performance between forced and suggested solutions on data with noise (Table S3), indicating that their reduced performance is likely due to their factorization approaches.

SigProfilerExtractor outperformed all other tools regardless of noise levels. Simulations with 5% noise reflect genomics datasets with ∼0.95 average sensitivity and precision of single base substitutions, similar to the recently published PCAWG cohort, which has 95% sensitivity (90% confidence interval, 88%–98%) and 95% precision (71%–99%).^43^ For WGS simulations with 5% noise, SigProfilerExtractor was able to identify between 20% and 50% more TP signatures while yielding more than 5-fold less FP signatures compared with the next seven best-performing tools (Figure 3C; Table S3).

To assess the ability of the top-performing tools to extract *de novo* mutational signatures from exome sequencing data, a WES benchmarking dataset, encompassing 20,000 unique synthetic cancer exomes, was generated by downsampling the WGS noise scenario with 5% noise. Exome data were challenging for all the *de novo* mutational signature extraction tools, resulting in a significant decrease in performance (Figure 3D). The average F_1_ score for all tools dropped from 0.61 for WGS simulations with 5% noise to 0.46 for WES simulations with 5% noise. Specifically, only SigProfilerExtractor showed an average F_1_ score above 0.60, with no other tool showing an F_1_ score above 0.53 (Figure 3D). SA was the only tool exhibiting an increased performance in WES compared with WGS in the 5% noise scenario, suggesting that the ARD approach was optimized for exome data (Table S5).

Lastly, simulations with 5% noise were additionally considered for benchmarking the different options provided by SigProfilerExtractor for performing *de novo* extraction. Specifically, we evaluated the effect of normalizing the input data (Gaussian mixture model [GMM], 100X, log2, and no normalization), the three different types of multiplicative updates for the NMF algorithm (Kullback-Leibler, Euclidean, or Itakura-Saito), and the two options for initializing the *S* and *A* matrices in the first step of the factorization: random initialization or nonnegative double singular vector decomposition (NNDSVD) initialization (STAR Methods). Overall, the objective function based on Kullback-Leibler updates outperformed the other two, independently of the normalization or initialization methods (Figure S2; Table S6). Regarding the four normalization methods, GMM, 100X, and log2 yielded comparable results, whereas running SigProfilerExtractor without previous transformation of the Poisson resampled matrix led to a significant drop in overall performance. The results obtained for the two different initialization methods, random and NNDSVD, differed depending on the other parameters. Nevertheless, they did not exhibit significant variations in the case of the top-performing NMF approach based on Kullback-Leibler updates and normalization using either GMM, 100X, or log2 transformation (Figure S2; Table S6).

### Reanalysis of 4,643 WGS and 19,184 WES human cancers

**Figure 4:**
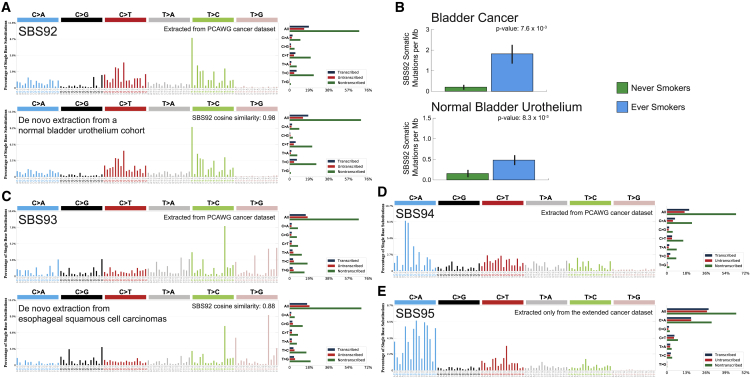
Novel signatures identified in a cohort of 4,643 WGS and 19,184 WES cancers Mutational signatures are displayed using 96 plots. Single base substitutions are shown using the six subtypes of substitutions: C>A, C>G, C>T, T>A, T>C, and T>G. Underneath each subtype are 16 bars reflecting the sequence contexts determined by the four possible bases 5′ and 3′ to each mutated base. Additional information whether mutations from a signature are in nontranscribed/intergenic DNA, on the transcribed strand of a gene, or on the untranscribed strand of the gene is provided adjacent to the 96 plots. (A) Mutational profile of signature SBS92 derived from the PCAWG cohort (top). Confirmation of the profile of signature SBS92 (bottom) by analysis of an independent WGS set of microbiopsies of histologically normal urothelium.^50^ (B) Bars are used to display average values for numbers of somatic substitutions per Mb attributed to signature SBS92 in bladder cancer and normal bladder urothelium. Green bars represent never smokers, whereas blue bars correspond to ever smokers. Error bars correspond to 95% confidence intervals. Each p value is based on a Wilcoxon rank-sum test. (C) Mutational profile of signature SBS93 derived from the PCAWG cohort (top). Confirmation of the profile of signature SBS93 (bottom) by analysis of an independent WGS set of esophageal squamous cell carcinomas.^43^ (D) Mutational profile of signature SBS94 derived from the PCAWG cohort. (E) Mutational profile of signature SBS95 derived only from liver hepatocellular carcinomas of the extended cohort. Signatures SBS94 and SBS95 were not identified in any additional independent cohort.

To demonstrate its ability to yield novel biological results, SigProfilerExtractor was applied to the recently published set of 2,778 WGS cancers from the PCAWG project.^43^ Additionally, we applied SigProfilerExtractor to an extended cohort of another 1,865 WGS and 19,184 WES cancers, encompassing data from The Cancer Genome Atlas (TCGA)^49^ as well as 261 other published studies and 35 different ICGC projects (Table S8). As previously done in our original PCAWG analysis of mutational signatures,^12^ extraction of mutational signatures was performed within each cancer type and across all samples (STAR Methods). In addition to all previously detected signatures,^12^ our direct application of SigProfilerExtractor revealed three novel mutational signatures in the PCAWG dataset: SBS92, SBS93, and SBS94. Further, a novel signature was also identified exclusively in the extended cohort: SBS95 (Figure 4; Table S7).

Signature SBS92 was found predominately in PCAWG bladder cancers; the signature was characterized by T>C mutations with strong transcriptional strand asymmetry consistent with damage on purines for all types of substitutions (Figure 4A). Signature SBS92 was 9-fold elevated (Figure 4B; p = 7.6 × 10^−3^; Wilcoxon rank-sum test) in bladder cancers of ever smokers compared with never smokers. An almost identical signature was identified by reanalyzing a recently published cohort of 88 WGS microbiopsies of histologically normal urothelium,^50^ with the similarity extending to both trinucleotide context and transcriptional strand asymmetry (Figure 4A; cosine similarity: 0.98; p < 10^−32^). Consistently, SBS92 was found to be 3-fold elevated in the normal urothelium of tobacco ever smokers compared with never smokers (Figure 4B; p = 8.3 × 10^−3^; Wilcoxon rank-sum test).

Signature SBS93 was identified almost exclusively in WGS stomach cancers, both from PCAWG^12^ and the extended cohort.^51^ SBS93 was characterized by T>C and T>G mutations with a strand asymmetry consistent with damage on pyrimidines for TpTpA contexts (mutated base underlined; Figure 4C). *De novo* extraction from the Mutographs cohort of 552 WGS esophageal squamous cell carcinomas,^52^ a cancer type not included in the PCAWG dataset,^43^ identified an analogous mutational signature, with the similarity extending to both trinucleotide context and transcriptional strand asymmetry (Figure 4C; cosine similarity: 0.88; p = 1.1 × 10^−6^). Signature SBS94 was found at high levels in a single colorectal PCAWG cancer, with smaller contributions to another eight colorectal cancers. The pattern of SBS94 was characterized by C>A mutations with a strand asymmetry indicative of damage on guanine (Figure 4D). Validation of somatic mutations by visual inspection confirmed that 98% of mutations contributed by SBS94 are likely real. Signatures SBS93 and SBS94 did not associate with any of the available PCAWG metadata,^43^ and their etiologies remain unknown. Signature SBS95 was only identified in a set of 109 WGS liver hepatocellular carcinomas from the extended cohort, with a profile characterized by C>A mutations and a bias toward the genic regions in comparison to the intergenic (Figure 4E). SBS95 was found as the predominant signature in five samples from the ICGC LINC-JP project, with modest contributions to another 24 samples. The lack of any associations or validations in external cohorts does not allow us to independently confirm signature SBS95, and, following our standard protocol, we have classified SBS95 as a possible artifactual signature.

## Discussion

The performed large-scale benchmarking demonstrates that SigProfilerExtractor outperforms 13 other tools for *de novo* extraction of mutational signatures for noiseless datasets as well as for datasets containing different levels of random noise, including synthetic data emulating WGS and WES cancers. Importantly, SigProfilerExtractor generates almost no FP signatures while still identifying a higher number of TP signatures when compared with any of the other tools (Figures 2D, 3C, and 3D). *De novo* extraction relies both on a factorization approach and on a model-selection algorithm for determining the total number of operative signatures (Figure 1). Benchmarking with forced model selection, where tools were required to extract the known number of ground-truth signatures, reveals that SigProfilerExtractor’s factorization performs better when compared with the factorizations of other tools (Figure 2B; Tables S1, S2, and S3). Similarly, benchmarking with suggested model selection, which most closely matches analysis of a real dataset, further demonstrates SigProfilerExtractor’s ability to reveal novel biological results (Figure 2A; Tables S1, S2, S3, S4, and S5). Interestingly, SigProfilerExtractor outperforms other tools when extracting correlated mutational signatures^53^ and signatures with overlapping profiles for specific contexts. In scenarios 5, 6, 9, and 10 (based on COSMIC signatures SBS2, SBS7a, and SBS7b, which share specific subtypes of C>T mutations), SigProfilerExtractor exhibited an average F_1_ score of 0.96, while the next best tools had F_1_ scores <0.90 (Table S1).

While our benchmarking evaluated 13 additional tools, 6 of the 13 tools internally rely on the same computational engine. Maftools, MutationalPatterns, MutSpec, SignatureToolsLib, SigMiner, and SomaticSignatures use the NMF R package^23^ to perform their factorization (Table 1), albeit with slightly different hyperparameters and, in some cases, distinct pre-processing of the input matrix. Predictably, these six tools have similar performance across many of the scenarios (Tables S1, S2, S3, S4, and S5). SigProfiler_PCAWG and MutSignatures utilize similar implementations of NMF.^26^ TensorSignatures makes use of the standard factorization algorithms included in TensorFlow.^41^ SigFit uses a previously developed nonnegative factorization method.^29^ In contrast, EMu, SA, SigneR, and SigProfilerExtractor provide original implementations of their factorization algorithms (Table 1). EMu was originally developed and tested on small datasets,^20^ and its benchmarking performance is perhaps unsurprising considering the large number of synthetic samples used in all scenarios. Surprisingly, the original implementations of SA and SigneR were susceptible to noise, yielding high numbers of FP signatures (Figure 3B).

While SigProfilerExtractor and SigProfiler_PCAWG, the latter used in the PCAWG analysis,^12^ share names, their computational engines are completely different. SigProfilerExtractor provides a fast-converging custom implementation of the multiplicative update algorithm,^16^ supporting three different objective functions and a GPU-based factorization implemented using PyTorch.^54^ In contrast, SigProfiler_PCAWG relies on a previously developed method by Brunet et al.^26^ for analysis of gene-expression data. SigProfilerExtractor supports automate noise-resistant selection of the matrix decomposition rank based on the Hungarian algorithm^45^ and the NMFk model selection approach,^37^ while SigProfiler_PCAWG does not provide an automate selection (Table 1). Importantly, SigProfilerExtractor also implements different normalization options preventing hypermutated tumors from skewing the factorization.

Seven of the tools did not provide an automatic approach for selecting the total number of signatures (Table 1). Instead, most of these tools offered methodologies for manual selection, thus, bringing user dependence and arbitrariness in selecting solutions. EMu, TensorSignatures, and SigneR automatically select the total number of signatures using Bayesian information criterion (BIC),^21^ while SA and SigMiner utilize ARD.^33^ SigFit’s selection approach is based on the Elbow method.^30^ SigProfilerExtractor leverages a modified version of the NMFk selection approach.^37^ Importantly, our simulations demonstrate that SigProfilerExtractor’s model selection is robust to noise, while the implemented BIC and ARD approaches are affected even by low levels of noise (Figure 3B).

High noise levels had limited effect on SigProfilerExtractor, causing the tool to miss some of the ground-truth signatures used to generate the synthetic datasets. Indeed, the average number of detected signatures dropped from 23.45 in the replicates without noise to 21.60 in those with 10% noise while maintaining a similar high precision (0.998 and 0.992, respectively; Figure 3B; Table S3). However, for other tools, the number of signatures either rose significantly with noise, leading to a notable increase in the FP signatures identified (MutationalPatterns, SA, and SigneR), or were kept stable but with a decrease in precision (MutSpec, SomaticSignatures, and SignatureToolsLib; Table S3). To deeply characterize the shape of the FP signatures identified by the different tools, we applied the Shannon equitability index to the results of the noise benchmark with suggested model selection (STAR Methods). Interestingly, the three tools showing a significant increase of FP signatures with noise (MutationalPatterns, SA, and SigneR) also showed a decrease in the Shannon equitability index (Table S4). In the case of SA, 5% noise reduced the average Shannon equitability for the FP signatures from 0.826 to 0.572, while 10% noise reduced it to 0.337 (in the range of the sparsest COSMIC signatures). This behavior was also found at lower levels for MutationalPatterns and SigneR. A similar trend was found for the average number of FP signatures detected, increasing with 10% noise from 4.40 to 90.95 for SA and from 2.50 to 19.70 and 6.15 to 20.85 for MutationalPatterns and SigneR, respectively (Table S3). These findings indicate that the higher the number of signatures detected in these tools, the higher the possibility to obtain more sparse FP signatures. On the other hand, the tools that maintain a similar number of detected signatures independently of the noise level (MutSpec, SomaticSignatures, and SignatureToolsLib) showed similar values for the Shannon equitability of their FP calls. In all cases, average values exceeded 0.88, indicating that mostly flat signatures are erroneously called by these tools (Table S4). In the case of SigProfilerExtractor, the average Shannon equitability of FP without noise and for all noise levels was, in all cases, over 0.92, following a similar trend as the previously mentioned tools. However, it is worth noting that only three FP signatures were detected by SigProfilerExtractor in all 20 replicates with 10% noise (600 total ground-truth signatures), whereas, for example, 417 and 1,819 were found for SigneR and SA, respectively (Table S3).

In addition to outperforming 13 other tools on simulated datasets, SigProfilerExtractor can reveal additional biological results, as demonstrated by identifying four novel signatures from the reanalysis of 23,827 sequenced cancers from the PCAWG and the extended datasets. Importantly, SigProfilerExtractor identified signature SBS92 (Figure 4), which is associated with tobacco smoking in WGS bladder cancers and in WGS microbiopsies from normal bladder urothelium. The strong transcriptional strand bias observed in SBS92 is indicative of an environmental mutagen exposure that damages purines. Tobacco smoke is a complex mixture of at least 60 chemicals,^11^ many capable of causing damage on purines. Interestingly, our and other prior analyses of exome-sequenced bladder cancers from TCGA^11^^,^^55^ did not reveal SBS92. Reanalysis of the set of TCGA bladder WES cancers^56^ with SigProfilerExtractor was also unable to detect SBS92 (STAR Methods). We suspect that the lack of SBS92 in the TCGA bladder cancers was due to the use of exome sequencing; note that SBS92 is predominately found in intergenic regions (Figure 4A), with most samples expected to have less than 15 mutations from SBS92 in their exomes. To confirm this hypothesis, we downsampled the WGS bladder cancers and the WGS microbiopsies from normal bladder urothelium to exomes. SigProfilerExtractor’s analysis of these downsampled genomes was unable to detect SBS92, confirming that exome sequencing is insufficient to identify signature SBS92 (STAR Methods).

In summary, here we report SigProfilerExtractor—a computational tool for *de novo* extraction of mutational signatures. We demonstrate that SigProfilerExtractor outperforms 13 other tools by conducting the largest benchmarking of bioinformatics approaches for extracting mutational signatures. Further, we apply SigProfilerExtractor to 4,643 WGS and 19,184 WES cancers and reveal four novel mutational signatures, including a signature putatively attributed to tobacco smoking mutagenesis in bladder cancer and in normal bladder epithelium.

### Limitations of the study

In this study, we assumed that mutational signatures are linearly and independently accumulating across the genomic landscape. While this assumption is likely correct for most signatures of small mutational events,^4^ such as substitutions and small insertions and deletions, it will be likely violated for signatures of larger mutational events including most copy-number signatures.^57^ In addition, a prior study has shown that the pattern of at least one substitution signature is not a superposition of individual alterations.^58^ Our current benchmarking ignores such scenarios, as they tend to be found in a small number of cancers with concurrent loss of both polymerase proofreading and mismatch repair.^58^ Lastly, this study focused on benchmarking the *de novo* extraction of mutational signatures from large sets of tumor samples, and it did not consider the assignment of signatures to a single cancer genome. Future benchmarking efforts will be required to evaluate the ability of different tools to accurately assign known mutational signatures to individual cancers.

## STAR★Methods

### Key resources table

**Table:** 

REAGENT or RESOURCE	SOURCE	IDENTIFIER
**Deposited data**		

PCAWG and extended dataset	263 published studies and 35 ICGC projects^6^^,^^43^^,^^49^^,^^51^^,^^59^^,^^60^^,^^61^^,^^62^^,^^63^^,^^64^^,^^65^^,^^66^^,^^67^^,^^68^^,^^69^^,^^70^^,^^71^^,^^72^^,^^73^^,^^74^^,^^75^^,^^76^^,^^77^^,^^78^^,^^79^^,^^80^^,^^81^^,^^82^^,^^83^^,^^84^^,^^85^^,^^86^^,^^87^^,^^88^^,^^89^^,^^90^^,^^91^^,^^92^^,^^93^^,^^94^^,^^95^^,^^96^^,^^97^^,^^98^^,^^99^^,^^100^^,^^101^^,^^102^^,^^103^^,^^104^^,^^105^^,^^106^^,^^107^^,^^108^^,^^109^^,^^110^^,^^111^^,^^112^^,^^113^^,^^114^^,^^115^^,^^116^^,^^117^^,^^118^^,^^119^^,^^120^^,^^121^^,^^122^^,^^123^^,^^124^^,^^125^^,^^126^^,^^127^^,^^128^^,^^129^^,^^130^^,^^131^^,^^132^^,^^133^^,^^134^^,^^135^^,^^136^^,^^137^^,^^138^^,^^139^^,^^140^^,^^141^^,^^142^^,^^143^^,^^144^^,^^145^^,^^146^^,^^147^^,^^148^^,^^149^^,^^150^^,^^151^^,^^152^^,^^153^^,^^154^^,^^155^^,^^156^^,^^157^^,^^158^^,^^159^^,^^160^^,^^161^^,^^162^^,^^163^^,^^164^^,^^165^^,^^166^^,^^167^^,^^168^^,^^169^^,^^170^^,^^171^^,^^172^^,^^173^^,^^174^^,^^175^^,^^176^^,^^177^^,^^178^^,^^179^^,^^180^^,^^181^^,^^182^^,^^183^^,^^184^^,^^185^^,^^186^^,^^187^^,^^188^^,^^189^^,^^190^^,^^191^^,^^192^^,^^193^^,^^194^^,^^195^^,^^196^^,^^197^^,^^198^^,^^199^^,^^200^^,^^201^^,^^202^^,^^203^^,^^204^^,^^205^^,^^206^^,^^207^^,^^208^^,^^209^^,^^210^^,^^211^^,^^212^^,^^213^^,^^214^^,^^215^^,^^216^^,^^217^^,^^218^^,^^219^^,^^220^^,^^221^^,^^222^^,^^223^^,^^224^^,^^225^^,^^226^^,^^227^^,^^228^^,^^229^^,^^230^^,^^231^^,^^232^^,^^233^^,^^234^^,^^235^^,^^236^^,^^237^^,^^238^^,^^239^^,^^240^^,^^241^^,^^242^^,^^243^^,^^244^^,^^245^^,^^246^^,^^247^^,^^248^^,^^249^^,^^250^^,^^251^^,^^252^^,^^253^^,^^254^^,^^255^^,^^256^^,^^257^^,^^258^^,^^259^^,^^260^^,^^261^^,^^262^^,^^263^^,^^264^^,^^265^^,^^266^^,^^267^^,^^268^^,^^269^^,^^270^^,^^271^^,^^272^^,^^273^^,^^274^^,^^275^^,^^276^^,^^277^^,^^278^^,^^279^^,^^280^^,^^281^^,^^282^^,^^283^^,^^284^^,^^285^^,^^286^^,^^287^^,^^288^^,^^289^^,^^290^^,^^291^^,^^292^^,^^293^^,^^294^^,^^295^^,^^296^^,^^297^^,^^298^^,^^299^^,^^300^^,^^301^^,^^302^^,^^303^^,^^304^^,^^305^^,^^306^^,^^307^^,^^308^^,^^309^^,^^310^^,^^311^^,^^312^^,^^313^^,^^314^^,^^315^^,^^316^^,^^317^^,^^318^^,^^319^^,^^320^^,^^321^^,^^322^	Table S8
Normal urothelium dataset	Lawson et al., 2020^50^	EGA: EGAD00001006113 and EGAD00001006116
Bladder urothelial carcinoma dataset	The Cancer Genome Atlas Research Network, 2014^56^	https://gdc.cancer.gov/
Synthetically generated dataset	This paper	Figshare: https://doi.org/10.6084/m9.figshare.20409430

**Software and algorithms**		

EMu 1.5.2	Fischer et al., 2013^20^	https://github.com/andrej-fischer/EMu
Maftools 2.2.0	Mayakonda et al., 2018^22^	https://bioconductor.org/packages/release/bioc/html/maftools.html
MutationalPatterns 3.0.1	Blokzijl et al., 2018^24^	https://bioconductor.org/packages/release/bioc/html/MutationalPatterns.html
MutSignatures 2.1.1	Fantini et al., 2020^25^	https://CRAN.R-project.org/package=mutSignatures
MutSpec 2.0	Ardin et al., 2016^27^	https://github.com/IARCbioinfo/mutspec
SigFit 2.0.0	Gori et al., 2020^28^	https://github.com/kgori/sigfit
SigMiner 1.0.0	Wang et al., 2020^31^	https://github.com/ShixiangWang/sigminer
SignatureAnalyzer	Kasar et al., 2015^32^; Taylor-Weiner et al., 2019^34^	https://github.com/broadinstitute/SignatureAnalyzer-GPU
SignatureToolsLib 0.0.0.9000	Degasperi et al., 2020^35^	https://github.com/Nik-Zainal-Group/signature.tools.lib
SigneR 1.16.0	Rosales et al.*,* 2016^36^	http://bioconductor.org/packages/release/bioc/html/signeR.html
SigProfiler_PCAWG (SigProExtractor) 0.0.5.48	Alexandrov et al., 2020^12^	https://pypi.org/project/sigproextractor/0.0.5.48/
SigProfilerExtractor 1.1.4	This paper	https://doi.org/10.5281/zenodo.6746540
SigProfilerExtractorR 1.1.0	This paper	https://doi.org/10.5281/zenodo.6941779
SigProfilerMatrixGenerator 1.2.4	Bergstrom et al., 2019^15^	https://github.com/AlexandrovLab/SigProfilerMatrixGenerator
SigProfilerSimulator 1.1.3	Bergstrom et al., 2020^48^	https://github.com/AlexandrovLab/SigProfilerSimulator
SomaticSignatures 2.26.0	Gehring et al., 2015^38^	https://bioconductor.org/packages/release/bioc/html/SomaticSignatures.html
SynSigGen	Alexandrov et al., 2020^12^	https://github.com/steverozen/SynSigGen
TensorSignatures 0.5.0	Vöhringer et al., 2021^40^	https://github.com/sagar87/tensorsignatures

**Other**		

Results from the benchmarking with synthetic datasets, including the appropriate input used to run each of the tools as well as the generated output	This paper	https://doi.org/10.6084/m9.figshare.20409430
Results from the benchmarking of the different options available in SigProfilerExtractor for matrix normalization, NMF initialization, and NMF objective function	This paper	https://doi.org/10.6084/m9.figshare.20411483
Results from the *de novo* extraction of mutational signatures from the Pan-Cancer Analysis of Whole Genomes (PCAWG) dataset	This paper	https://doi.org/10.6084/m9.figshare.20406279
Results from the *de novo* extraction of mutational signatures from the extended dataset	This paper	https://doi.org/10.6084/m9.figshare.20406326
Summarized collection of all input mutational matrices, as well as *de novo* extracted mutational signatures and activities for both PCAWG and extended datasets	This paper	https://doi.org/10.6084/m9.figshare.20293890
Results from the *de novo* extraction of mutational signatures for confirming the patterns of the novel signatures for additional datasets	This paper	https://doi.org/10.6084/m9.figshare.20406156
Results from the *de novo* extraction of mutational signatures from downsampling of whole-genome sequenced samples to whole-exomes	This paper	https://doi.org/10.6084/m9.figshare.20406276
Resource website for the COSMIC reference set of mutational signatures	Tate et al., 2019^42^	https://cancer.sanger.ac.uk/signatures/

### Resource availability

#### Lead contact

Further information and requests should be directed to and will be fulfilled by the lead contact, Ludmil B. Alexandrov (l2alexandrov@health.ucsd.edu).

#### Materials availability

This study did not generate new unique reagents.

### Experimental model and subject details

No experimental models were utilized as part of this publication. No novel subjects were collected as part of this publication.

### Method details

#### Computational implementation of SigProfilerExtractor and its seven modules

The implementation of SigProfilerExtractor can be separated into seven distinct modules which are packaged together into a single bioinformatics tool. *Module 1* processes the initial input data, which can be provided as either a mutational catalog containing a set of somatic mutations or a mutational matrix. *Module 2* is responsible for resampling and normalization of the mutational matrix prior to performing nonnegative matrix factorization. *Module 3* performs matrix factorization using nonnegative matrix factorization with multiple replicates. *Module 4* utilizes custom clustering to derive consensus solutions and to perform model selection. *Module 5* decomposes the derived set of *de novo* signatures to a set of previously derived COSMIC signatures. *Module 6* is responsible for calculating the activities of different signatures in individual samples. *Module 7* handles the extensive outputting and plotting of the different analysis performed by SigProfilerExtractor. In principle, each of these modules allows extensive customization. SigProfilerExtractor provides a seamless integration of these seven modules that allows using them in an orchestrated and preconfigured manner with little input from a user.

##### Module 1: Processing of input mutational catalogs or input mutational matrices

SigProfilerExtractor deciphers mutational signatures from a mutational matrix ***M*** with *t* rows and *n* columns; rows represent mutational channels while columns reflect individual cancer samples (Figure 1A). The value of each cell in the matrix, ***M***, corresponds to the number of somatic mutations from a particular mutational channel in each sample. The mutational matrix can be provided as a text file with the first column containing the names of the mutational channels and the first row containing the names of the examined samples, thus supporting nonnegative matrix factorization for any custom matrix dataset. Alternatively, users can provide a mutational catalog of somatic mutations in a commonly used format (*e.g.,* VCF, MAF, *etc.*) and this mutational catalog will be internally converted into the appropriate mutational matrix by SigProfilerMatrixGenerator.^15^

##### Module 2: Resampling of the input mutational matrix and normalizing the resampled matrix

SigProfilerExtractor does not factorize the original input matrix. Rather, prior to performing matrix factorization, SigProfilerExtractor performs independent Poisson resampling of the original matrix for each replicate.^4^ As such, the matrix factorized in each replicate is never the same for a given value of *k* (Figure 1B). The resampling is performed to ensure that Poisson fluctuations of the matrix do not impact the stability of the factorization results. Additional normalization is performed after resampling to overcome potential skewing of the factorization from any hypermutators. SigProfilerExtractor supports four standard normalization methods^323^: *(i)* Gaussian mixture model (GMM) normalization (default); *(ii)* 100X normalization; *(iii)* log2 normalization; *(iv)* no normalization. *No normalization* does not perform any additional transformation on the Poisson resampled matrix. In *log2 normalization*, the sum of each column in the matrix is derived and logarithm with base 2 is calculated for each of these sums. Each cell in a column of the matrix is multiplied by the log2 of the column-sum and subsequently divided by the original column sum. In *100X normalization*, the sum of each column in the matrix is derived. For each column where the sum exceeds 100 times the number of mutational channels (*i.e.*, 100 times the number of rows in the matrix), each cell in the column is multiplied by the 100 times the number of mutational channels and subsequently divided by the original column sum. This normalization ensures that no sample has a total number of mutations above 100 times the number of mutational channels. *GMM normalization* encompasses a two-step process. The first step derives the normalization cutoff value in a data-driven manner using a Gaussian mixture model (GMM). The second step normalizes the appropriate columns using the derived cutoff value. The first step uses a GMM to separate the samples into two groups based on their total number of mutations; the total number of mutations in a sample reflects the sum of a column in the matrix. The group with larger number of samples is subsequently selected, and the same process is applied iteratively until it converges. Convergence is achieved when the mean of the two groups is separated by no more than four standard deviations of the larger group. A cutoff value is derived as the average value plus two standard deviations from the total number of somatic mutations in the last large group. If the derived cutoff value is below 100 times the number of mutational channels, the cutoff value is adjusted to 100 times the number of mutational channels. For each column where the sum exceeds the derived cutoff value, each cell in the column is multiplied by the cutoff value and subsequently divided by the original column sum. Note that 100X normalization is performed if the means of the first two groups are not separated by at least four standard deviations. In all cases, fractional values after normalization are used as input for the factorization, and columns with a sum of zero, reflecting genomes without any somatic mutations, are ignored to avoid division by zero.

##### Module 3: Matrix factorization using nonnegative matrix factorization with replicates

By default, SigProfilerExtractor factorizes the matrix ***M*** with different ranks searching for an optimal solution between *k =* 1 and *k =* 25 mutational signatures. For each value of *k*, by default, the tool performs 100 independent nonnegative matrix factorizations of the normalized Poisson resampled input matrices. Thus, for each value of *k*, SigProfilerExtractor generates 100 distinct factorizations of normalized Poisson resampled matrices resulting into 100 different matrices ***S***, each matrix reflecting the patterns of the *de novo* mutational signatures, and 100 different matrices ***A***, each matrix reflecting the activities of the *de novo* mutational signatures (Figure 1B). To perform each of these factorizations, SigProfilerExtractor utilizes a custom implementation of the multiplicative update algorithm.^16^ Specifically, SigProfilerExtractor initializes the ***S*** and ***A*** matrices in the first step of the factorization using either random initial conditions (default) or one of the derivatives of nonnegative double singular vector decomposition.^324^ SigProfilerExtractor provides internal support for minimizing three different objective functions based on: *(i)* generalized Kullback-Leibler updates (default); *(ii)* Euclidean updates; *(iii)* Itakura-Saito updates. By default, the tool performs all factorizations using multithreading of central processing units (CPUs) and provides support for factorization using graphics processing units (GPUs) by leveraging PyTorch.^54^ In all cases, by default, the implemented minimization performs at least 10,000 iterations (also known as NMF updates or NMF multiplicative update steps) with a maximum of 1,000,000 iterations. By default, the convergence tolerance of the algorithm is set to 10^−15^. Note that SigProfilerExtractor allows reconfiguring all factorization parameters.

##### Module 4: Custom partition clustering and performing model selection

The previously described *Module 3* generates a number of sets with each set containing, by default, 100 different matrices ***S***, where each matrix reflects the patterns of *de novo* mutational signatures for a particular factorization of a normalized Poisson resampled matrix. One set, containing 100 different matrices ***S***, is generated for each of the interrogated total number of operative signatures, *k*, with a default range for *k* between 1 and 25 signatures. For each value of *k*, *Module 4* first performs custom clustering of the ***S*** matrices and, subsequently, applies a modified version of the NMFk model selection approach to select the optimal value of *k*^51^ (Figure 1B). Specifically, for each value of *k*, the clustering is initialized with *k* random centroids. One of the ***S*** matrices is randomly chosen, and its columns matched to the most similar centroids with no two columns assigned to the same cluster. The process is repeated until the columns of all ***S*** matrices in the set are assigned to their respective clusters. SigProfilerExtractor implements the Hungarian algorithm^45^ to pair consensus vectors from two matrices (*i.e.,* cluster centroids and mutational signature from a matrix ***S***); the Hungarian algorithm maximizes the total cosine similarities of all paired vectors between two matrices.^45^ After assigning all columns to a cluster, the centroid of each cluster is recalculated by evaluating the average of all columns/vectors in a cluster. This process continues iteratively until the average silhouette coefficient converges (*i.e.,* its value does not change by more than 10^−12^). After convergence for a given value of *k*, the centroids of the clusters are reported as consensus mutational signatures, an overall reconstruction error is calculated for describing the original input matrix, ***M***, and stability is calculated for each signature by computing the silhouette value^325^ of the cluster corresponding to that signature (Figure 1B). The silhouette value of a cluster measures the similarities of the objects assigned to that cluster compared to any other cluster. Silhouette values range from −1.0 to +1.0 with values above zero indicating that, on average, objects have a higher similarity with their own cluster compared to their nearest clusters. Note that signatures with low stability correspond to a lack of uniqueness of the NMF due to Poisson resampling and/or to the potential existence of multiple convergent stationary points in the NMF solution.^47^

Our custom clustering is performed for each of the interrogated total number of operative signatures, *k*, with a default range for *k* between 1 and 25 signatures. After performing clustering, for each value of *k*, one has derived: *(i)* the consensus set of mutational signatures; *(ii)* an overall reconstruction error for describing the original input matrix; and *(iii)* stability value for each of the identified consensus mutational signatures.

SigProfilerExtractor performs a solution selection based on the stability of signatures in a solution and the ability of these signatures to reconstruct the original input matrix. By default, for whole-genome sequenced samples, SigProfilerExtractor will consider solutions stable if the signatures derived in the solution have an average stability above 0.80 with no individual signature having stability below 0.20 (0.70 and 0.10, respectively, are the recommended thresholds for extractions based on whole-exome sequenced samples). To reduce overfitting, the tool also measures the information gained from the extracted set of signatures in each solution. SigProfilerExtractor compares, using Wilcoxon rank-sum tests, the reconstruction errors across all samples from the stable solution with the greatest number of signatures to the reconstruction errors across all samples from stable solutions with lower number of signatures. Stable solutions with lower number of signatures are compared in a decreasing order to their total number of signatures with comparison stopping if the Wilcoxon rank-sum test yields a p *value* below 0.05 (*i.e.*, reflecting that a solution does not describe the original data as good as the stable solution with the greatest number of signatures). The stable solution with the lowest number of signatures and a Wilcoxon rank-sum test p *value* above 0.05 is selected as the optimal solution. If no solution has a Wilcoxon rank-sum test p *value* above 0.05, the stable solution with the greatest number of signatures is selected as the optimal solution. This test is not considered when extracting signatures from whole-exome sequenced samples, to favor sensitivity in low-mutation-count data. Note that while SigProfilerExtractor selects an optimal solution, it outputs all the information necessary to evaluate mutational signatures and their activities for all other stable and unstable solutions.

##### Module 5: Decomposing *de novo* extracted signatures to known COSMIC signatures

SigProfilerExtractor provides a module for decomposing each of the *de novo* extracted mutational signatures to a set of previously derived signatures. By default, the tool decomposes each of the signatures in the optimal solution to a set of COSMICv3.1 reference signatures^12^ with support for signatures of single base substitutions (SBS), doublet base substitutions (DBS), and small insertions and deletions (ID). Since the SBS COSMICv3.1 reference signatures were derived under the SBS-96 classification,^15^ any extended classification of single base substitutions (*e.g.*, SBS-288 and SBS-1536)^15^ is first collapsed to the SBS-96 classification and, subsequently, decomposed to the COSMICv3.1 reference signatures.^12^ The decomposition functionality leverages the nonnegative least squares (NNLS) algorithm^326^ as its main computational engine. A mixture of addition and removal steps (add-remove functionality) were developed to estimate the list of COSMIC signatures for a *de novo* signature. Specifically, for each *de novo* signature, a COSMIC signature is iteratively added to a list of signatures used to explain the *de novo* signature, NNLS is applied, and the signature which addition causes the greatest decrease of the L2 error is selected. If this greatest decrease is more than a specific threshold (default value of 5%) then the signature is included in the list of signatures used to explain the *de novo* signature. The addition is immediately followed by a removal step. Each COSMIC signature in the list of signatures used to explain the *de novo* signature are iteratively removed, NNLS is applied, and the signature that causes the least decrease of the L2 error is selected. If this least decrease is less than a specific threshold (default value of 1%) then the signature is removed from the list of signatures used to explain the *de novo* signature. The addition and removal steps are iterated until no signature is added or removed from the list of signatures used to explain the *de novo* signature. Several previously implemented rules for mutational signatures are incorporated by default in the decomposition module.^12^ Specifically, for signatures of single base substitutions: *(i)* the list of signatures used to explain the *de novo* signature is initialized with the clock-like signatures SBS1 and SBS5;^9^ *(ii)* biologically connected signatures are included as previously done in Ref ^12^ (*e.g.,* if SBS17a is included in the list then SBS17b is also included the list). The decomposition module is highly customizable as it allows changing all default parameters as well as adding additional new rules or removing existing rules for inclusion and exclusion of particular signatures.

##### Module 6: Evaluating activities of mutational signatures in individual samples

*De novo* extracted and COSMIC derived signatures are refitted to individual samples using nonnegative least squares (NNLS).^326^ *Module 6* internally utilizes the add-remove functionality of *Module 5* with each sample in the original matrix, ***M***, being individually examined. For *de novo* mutational signatures, all *de novo* signatures are initially added to the list of signatures used to explain the sample and a removal step with a cutoff of 2% is applied. To assign COSMIC signatures in a sample, the module first derives the set of *de novo* signatures in that sample. Decomposition to the COSMICv3.1 signatures using *Module 5* is performed for each of the *de novo* signatures and the identified COSMICv3.1 signatures are refitted using the add-remove functionality with a removal and addition cutoffs set at 5%. Finally, the activity matrix is constructed by combining the activity vectors generated for all samples in the dataset.

##### Module 7: Outputting and plotting of analysis results

All previous modules make use of *Module 7* for outputting and plotting of the generated results. It should be noted that SigProfilerExtractor provides extensive output for the interrogated total number of operative signatures, *k*, with a default range of *k* between 1 and 25 signatures. For each value of *k*, SigProfilerExtractor outputs the set of operative *de novo* mutational signatures, the activities of the operative signatures, and an extensive set of information related to individual samples, individual *de novo* signatures, and the overall convergence of the factorization and clustering. *Module 7* also provides additional information when run in debug mode. In addition to outputting information, SigProfilerExtractor also generates a bouquet of plots both for each value of *k* as well as for the suggested optimal solution. SigProfilerExtractor utilizes all previously implemented plots in SigProfilerPlotting^15^ as well as includes several newly developed visualizations.

#### Analysis of the genomics data from cancer and normal somatic tissues

For all examined whole-genome sequenced cancer and normal somatic tissues, *de novo* extraction of mutational signatures was performed with SigProfilerExtractor with default parameters using two distinct mutational classifications: SBS-96 and SBS-288. Only the SBS-96 classification was used for whole-exome sequenced data. The SBS-96 mutation classification incorporates the six types of single base substitutions: C>A, C>G, C>T, T>A, T>C, and T>G. Each type of single base substitution is further separated into 16 subtypes determined by the four possible bases 5′ and -3′ adjacent to each mutated base. The SBS-288 mutation classification extends the SBS-96 mutation classification by adding additional information for each of the 96 subtypes. Specifically, SBS-288 incorporates whether a single base substitution is in non-transcribed/intergenic DNA, on the transcribed strand of a gene, or on the untranscribed strand of the gene. *De novo* extraction was performed separately for all examined datasets. Specifically, SigProfilerExtractor was applied: *(i)* to all 2,778 whole-genome sequenced cancers from the Pan-Cancer Analysis of Whole Genomes project^43^; *(ii)* to all 1,865 whole-genome and 19,184 whole-exome sequenced cancers from the extended cohort (Table S8); *(iii)* to all samples in each of the 37 cancer types of the Pan-Cancer Analysis of Whole Genomes project^43^ with each cancer type examined separately; *(iv)* to all samples in each of the 66 cancer types of the extended cohort (Table S8) with each cancer type examined separately; *(v)* to all 88 whole-genome sequenced microbiopsies of histologically normal urothelium^50^; *(vi)* to the complete set of whole-genome sequenced bladder cancers from TCGA^56^; *(vii)* to exome downsampling of all bladder whole-genome sequenced cancers from the Pan-Cancer Analysis of Whole Genomes project^43^; *(viii)* to exome downsampling of all 88 whole-genome sequenced microbiopsies of histologically normal urothelium.^50^ In all cases, the mutational catalog of each sample was taken from the respective original publications. The results from all performed *de novo* extractions can be found at: ftp://alexandrovlab-ftp.ucsd.edu/pub/publications/Islam_et_al_SigProfilerExtractor/. Downsampling of whole-genome sequenced samples to whole-exome was performed using SigProfilerMatrixGenerator.^15^

#### Additional approaches for miscellaneous analysis

Cosine similarity was used to compare the profiles of different mutational signatures. P-values can be attributed to cosine similarities based on a null hypothesis of uniform random distribution of nonnegative vectors.^48^

Briefly, the prevalence of somatic mutations in a whole-exome sample was calculated based on the identified mutations in protein coding genes and assuming that an average whole-exome has sufficient coverage of 30.0 megabase-pairs in protein coding genes. The prevalence of somatic mutations in a whole-genome sample was calculated based on all identified mutations and assuming that an average whole-genome has sufficient coverage of 3.00 gigabase-pairs.

In order to characterize the shape of the false positive signatures identified by the different signature extraction tools, we used the Shannon equitability index metric^327^ for mutational signatures, defined as follows.

$$Shannon\;equitability\;index=-\frac{\sum_{i=1}^{t}p_{i}\ln\;p_{i}}{\ln\;t}$$

In this formula, ***p*** represents the probability of a mutation caused by a specific mutational signature to belong to a specific mutational channel, whereas ***t*** is the total number of mutational channels or rows of the input mutational matrix ***M*** (which corresponds to 96 in the case of the well-known SBS-96 classification). The range of the Shannon equitability index goes from zero, characterizing a trivial signature where only one specific mutational channel is possible (*i.e.*, null diversity of mutational channels), to one, which would correspond to a completely uniform signature where all mutational channels accumulate the same probability (for example, 1/96 in the case of the SBS-96 classification). Indeed, well-known COSMIC signatures commonly defined as sparse, such as clock-like SBS1 or APOBEC-related SBS2 show a Shannon equitability index of 0.409 and 0.267, respectively, whereas signatures usually defined as flat, including SBS3, SBS5, and SBS40 display a much higher Shannon equitability of 0.961, 0.941, and 0.949, respectively, which is closer to the maximum value.

#### Creation of scenarios with synthetic datasets

Benchmarking was performed on simulated datasets with and without noise. These synthetic datasets were created using a previously described method.^12^ All datasets without noise were categorized as different scenarios with many of these scenarios attempting to emulate a particular set of cancer types. Specifically, 20 scenarios were created for the SBS-96 mutational classification and 12 additional scenarios were generated for the extended number of channels. For the SBS-96 classification, COSMIC signatures originally extracted from the PCAWG dataset^12^ as well as signatures extracted using SignatureAnalyzer^12^ and random signatures were used as ground-truth signatures. Many of the scenarios were created using a combination of tissue specific signatures. Signature profiles of extended scenarios (E) were based either on random signatures or on composite signatures extracted by SigProfiler_PCAWG or SignatureAnalyzer. Composite signatures consist of a total of 1,697 mutation types, encompassing an amalgamation of: 1536 strand-agnostic single base substitutions (SBS-1536) in a pentanucleotide context, 78 doublet-base substitutions (DBS-78), and 83 types of small insertions and deletions (ID-83).

Attributions of signatures in the different scenarios associated with a cancer type, *t*, were generated based on three parameters that were in turn based on the observed statistics for each signature, *s*, in cancer type *t*: π, the proportion of tumors of cancer type *t* with signature *s*; μ, the mean of log_10_ of the number of *s* mutations across those tumors of type *t* that have signature *s*; and σ, the standard deviation of log_10_ of the numbers of *s* mutations across those *t* tumors that have *s*.

Synthetic scenarios were labeled as easy, medium, and hard based on the number of operative signatures in each scenario. Based on our most recent analysis of mutational signatures in 82 cancer types,^12^ approximately 7.4% of human cancer types have 5 or less signatures (reflected in simulations of easy scenarios), 15.9% have 11 to 21 signatures (medium scenarios), and 59.5% have 25 or more signatures (hard scenarios). Note that 17.2% of cancer types have either between 5 and 10 signatures or between 22 and 24 signatures.

Detailed description of each of the used scenarios for benchmarking is provided below. Note that some of the generated scenarios were initially created as part of Ref. ^12^. The computational approach for generating the synthetic data can be found at: https://github.com/steverozen/SynSigGen. Noiseless scenarios 1 to 14 had only a single replicate while scenario 15 through 20 had 10 replicates each, and WGS and WES noise scenarios had 20 replicates each.

##### Scenarios 1, 2, E-1, and E-2

The scenarios were generated to emulate a subset of the pancreatic adenocarcinoma PCAWG dataset with a total 1,000 synthetic samples. Ground-truth signatures were based on COSMIC as well as on signatures extracted by SignatureAnalyzer.

##### Scenarios 3, 4, E-3, and E-4

Mutational spectra generated from combinations of flat, relatively featureless mutational signatures. A total of 1,000 synthetic tumors emulating a mixture of 500 synthetic renal cell carcinomas (high prevalence and mutation load from SBS5 and SBS40 signatures) and 500 synthetic ovarian adenocarcinomas (high prevalence of and mutation load from SBS3). Ground-truth signatures were based on COSMIC and signatures extracted by SignatureAnalyzer. This dataset embodies tumors with high prevalence of the main flat signatures, SBS3, SBS5, and SBS40, in a realistic context.

##### Scenarios 5, 6, E-5, and E-6

Mutational spectra generated from signatures with overlapping and potentially interfering profiles. A total of 1,000 synthetic tumors composed mostly from SBS2, SBS7a, and SBS7b. Mutational load distributions were drawn from bladder transitional cell carcinoma (SBS2) and skin melanoma (SBS7a, SBS7b). Ground-truth signatures were based on COSMIC and signatures extracted by SignatureAnalyzer. Most spectra contain both signatures SBS7a and SBS7b. The potential interference is between SBS2 (mainly C>T) and SBS7a, SBS7b (mainly C>T).

##### Scenarios 7, 8, E-7, and E-8

Mutational spectra generated from combinations of flat, relatively featureless mutational signatures. A total of 1,000 synthetic tumors emulating a mixture of 500 synthetic renal cell carcinomas (high prevalence and mutation load from SBS5 and SBS40 signatures) and 500 synthetic ovarian adenocarcinomas (high prevalence and mutation load from SBS3). Ground-truth signatures were based on COSMIC and signatures extracted by SignatureAnalyzer. This dataset embodies tumors with high prevalence of the main flat signatures, SBS3, SBS5, and SBS40, in a simplified fashion, where only these three signatures are present.

##### Scenarios 9, 10, E-9, and E-10

Mutational spectra generated from signatures with overlapping and potentially interfering profiles. A total of 1,000 synthetic tumors composed from SBS2, SBS7a, and SBS7b. Mutational load distributions were drawn from bladder transitional cell carcinoma (SBS2) and skin melanoma (SBS7a, SBS7b). Ground-truth signatures were based on COSMIC and signatures extracted by SignatureAnalyzer. Most spectra contain both groups of signatures. The potential interference is between SBS2 (mainly C>T) and SBS7a, SBS7b (mainly C>T). This dataset presents synthetic tumors containing these three signatures in a simplified fashion, excluding the presence of any additional mutational signature.

##### Scenarios 11, 12, E-11, and E-12

A set of 30 random mutational signature profiles based on SBS-96 classification and a set of 30 random 1,697 feature signature profiles (mimicking SignatureAnalyzer’s composite signatures). Each of these sets of random signatures were used in two types of exposures, one with more (mean ∼15.6) signatures per tumor and one with fewer (mean ∼4) signatures per tumor.

##### Scenarios 13 and 14

A set of 2,700 synthetic whole-genome samples with mutational spectra matching the ones observed in PCAWG, including 300 spectra from each of 9 different cancer types. These spectra consist of 300 synthetic spectra from each of the following cancer types: bladder transitional cell carcinoma, esophageal adenocarcinoma, breast adenocarcinoma, lung squamous cell carcinoma, renal cell carcinoma, ovarian adenocarcinoma, osteosarcoma, cervical adenocarcinoma, and stomach adenocarcinoma. Ground-truth signatures were based on COSMIC as well as on signatures extracted by SignatureAnalyzer.

##### Scenarios 15 and 18

A set of 5 random mutational signature profiles based on SBS-96 mutational classification. A total of 200 synthetic tumors were generated with one scenario containing an average of 3 signatures per tumor while the other scenario had an average of 5 signatures per tumor.

##### Scenarios 16 and 19

A set of 15 random mutational signature profiles based on SBS-96 mutational classification. A total of 200 synthetic tumors were generated with one scenario containing an average of 10 signatures per tumor while the other scenario had an average of 5 signatures per tumor.

##### Scenarios 17 and 20

A set of 25 random mutational signature profiles based on SBS-96 mutational classification. A total of 200 synthetic tumors were generated with one scenario containing an average of 15 signatures per tumor while the other scenario had an average of 5 signatures per tumor.

##### WGS noise scenario

In addition to noiseless scenarios, we simulated 20 replicates of a WGS scenario with noise: 10 of the replicates were based on scenario 11 and another 10 replicates were based on scenario 12. In each case, white Gaussian noise was added to each replicate in order to study the performance of the tools at different amounts of noise, emulating differences in the sequencing quality of real datasets. Specifically, random noise was introduced for different noise levels (0%, 1%, 2.5%, 5%, or 10%) by resampling every data point in the mutational matrix (*i.e.*, reflecting the number of mutations of a specific mutation type in a cancer sample) using a Gaussian distribution where the mean corresponds to the value of the data point, and the standard deviation is the value of the data point multiplied by the specific noise level. Subsequently, decimal values were truncated, and negative values were replaced with zeros. Overall, 5 distinct levels of noise were generated, each repeated 20 times, with an average noise level corresponding to 0%, 1%, 2.5%, 5%, and 10% of all mutations observed in the replicate.

##### WES noise scenario

A WES-based scenario was generated by downsampling the WGS-based noise scenario corresponding to a 5% noise level, reflecting high-quality genomic datasets. The downsampling of synthetic cancer genomes and randomly generated ground-truth mutational signatures was done in a two-step process. Firstly, WGS-based SBS-96 mutational matrices were simulated with SigProfilerSimulator^48^ to obtain VCF files with simulated synthetic mutations spanning across the whole genome. Subsequently, exome-specific SBS-96 mutational matrices were created with SigProfilerMatrixGenerator^15^ including exclusively the synthetic mutations affecting the exome portion of the human genome based on the SureSelect Human All Exon v7 protocol (Agilent, Santa Clara, CA, USA). This two-step process allows considering the differences in trinucleotide frequencies between the exome and the whole human genome, which would not be captured by direct downsampling of the original WGS mutational matrices based on the fact that the exome constitutes ∼2% of the human exome but has a different trinucleotide context.

#### Benchmarking bioinformatic tools for *de novo* extraction of mutational signatures

The hitherto described synthetic scenarios were used to compare SigProfilerExtractor and thirteen other tools for *de novo* extraction of mutational signatures. The method and parameters used to extract signatures from the simulated datasets using each tool are described below. With the exception of EMu and SignatureAnalyzer, which support only detection of the total number of mutational signatures without a prespecified range, all other tools required specifying the range for the total number of operative mutational signatures. The ranges for benchmarking with suggested model selection, which most closely matches the analysis of a real dataset with unknown number of signatures, are provided in Table S9 for each of the scenarios. Benchmarking with forced model selection, where tools were required to extract the known number of ground-truth mutational signatures, performed *de novo* extraction based on the ground-truth number of total mutational signatures (Table S9). For the WGS noise scenario, the same ranges used for noiseless scenarios 11 and 12 were applied. In the case of the WES noise scenario, a reduced range of signatures was used to optimize running time, based on the low sensitivity observed for all tools on WES data compared to WGS. This WES-specific range was used to extract *de novo* mutational signatures in all tools except for SigneR, whose signature selection method depends on the maximum number of signatures tested (original WGS range was applied).

##### SigProfilerExtractor

The default settings of SigProfilerExtractor (version 1.1.4) were used to extract mutational signatures with minor modifications to reduce overall extraction time. Specifically, we utilized “NMF replicates” = 100, “minimum NMF iterations” = 1,000, “maximum NMF iterations” = 200,000, “NMF test convergence” = 1,000 and “NMF tolerance” = 1e-08 parameter settings for all scenarios without noise and all WGS and WES replicates with noise.

##### SigProfiler_PCAWG

The default settings of SigProExtractor^12^ (version 0.0.5.48) were used to extract signatures from the benchmark scenarios with exception of “totaliteration” = 100.

##### SignatureAnalyzer

For the scenarios with and without noise, we used the default parameters for SignatureAnalyzer^32^^,^^34^ described in https://github.com/broadinstitute/SignatureAnalyzer-GPU. For the extended scenarios, the CPU version of SignatureAnalyzer was used with 20 runs and default parameters. The mode number of signatures counts was selected for further evaluation.

##### MutationalPatterns

We downloaded MutationalPatterns^24^ version 3.0.1 according to the instructions at https://bioconductor.org/packages/release/bioc/html/MutationalPatterns.html. To extract signatures, we used the NMF method with default parameters with the exception of the “nrun” parameter. The “nrun” parameter was set to 200 in order to increase the reliability of the extraction. To select the optimum number of signatures, as suggested by the developers of the tool, we used the RSS plot that is generated in the NMF rank survey plot.

##### SignatureToolsLib

We downloaded SignaturesToolsLib^35^ from https://github.com/Nik-Zainal-Group/signature.tools.lib and the tool was used using the parameters recommended by the authors. Specifically, we utilized “nboots” = 20, “nrepeats” = 200 and “filterBest_RTOL” = 0.001. As suggested by the developers, we selected the optimum number of signatures from the plot illustrating the overall metrics. We mostly used the “norm.Error” and “Ave.SilWid” with Clustering with Matching (MC) to select the total number of operative mutational signatures.

##### SigneR

We used the SigneR^36^ version 1.16.0 as described in http://bioconductor.org/packages/release/bioc/vignettes/signeR/inst/doc/signeR-vignette.html. The signatures were extracted with default parameters without using an opportunity matrix.

##### MutSpec

We used the command line platform of MutSpec^27^ version 2.0, as described at https://github.com/IARCbioinfo/mutspec. To extract signatures, we used the default parameters. As suggested by the developers, we estimated the optimum number of signatures from the “NMF rank survey” plot generated from the “MutSpec-NMF_Estimate_Signatures” module of the package.

##### SomaticSignatures

We followed the instructions described at https://www.bioconductor.org/packages/release/bioc/vignettes/SomaticSignatures/inst/doc/SomaticSignatures-vignette.html to use the NMF method to assess and extract mutational signatures. SomaticSignatures^38^ version 2.26.0 was used with default parameters. To access the number of signatures, we increased the value of “nmf_replicates” from 5 to 30 in order to get better reproducibility. As suggested by the developers, we selected the optimum number of signatures using the “plotNumberSignatures” function provided by the tool. In the plots, we relied on the RSS and explained variance value to choose the optimum solution.

##### Maftools

We followed the instructions provided at https://www.bioconductor.org/packages/release/bioc/vignettes/maftools/inst/doc/maftools.html to download and extract signatures using Maftools^22^ version 2.2.0. As suggested by the developers, we estimated the goodness of fit to decide the optimal number of signatures using the “estimateSignatures” function. Then we extracted the corresponding optimal number of signatures using the “extractSignatures” function provided by the tool. All settings were kept as default, except we increased the value of “nTry” from 6 to 20 in order to increase reproducibility.

##### SigMiner

We installed SigMiner^31^ version 1.0.0 according to the instructions provided at https://shixiangwang.github.io/sigminer-doc/. All the parameters were set as defaults to both estimate as well as extract mutational signatures. To select the optimum number of signatures, as suggested by the developers, we assessed the statistics provided in the NMF rank survey plot.

##### SigFit

We used the instructions provided at http://htmlpreview.github.io/?https://github.com/kgori/sigfit/blob/master/doc/sigfit_vignette.html to download and extract signatures from SigFit^28^ version 2.0.0. Signature extraction was done using default parameters with the exception of the total number of iterations which was set at 100.

##### EMu

Benchmarking for EMu^20^ was done with version 1.5.2 using default parameters for suggested extractions. Additionally, the optional “force” parameter was used for benchmarks done with a specific number of signatures. EMu was the only tool that was unable to complete *de novo* extractions from a number of synthetic datasets (Tables S1, S2 and S3) with the tool either running out of memory on instances with 256 GiB memory or running for 4+ weeks without producing any results. These scenarios were considered as failed and assigned F_1_ scores of zero.

##### MutSignatures

MutSignatures^25^ version 2.1.1 was downloaded and run according to https://cran.r-project.org/web/packages/mutSignatures/vignettes/get_sarted_with_mutSignatures.html. Signatures were extracted using 500 iterations (“num_totIterations” = 500).

##### TensorSignatures

TensorSignatures^40^ version 0.5.0 was downloaded and run according to the instructions at https://github.com/sagar87/tensorsignatures. Input VCFs were generated from the matrices by running SigProfilerSimulator.^48^ The headers of the VCF files were modified for TensorSignatures to compute the trinucleotide normalization. TensorSignatures was applied using 10,000 epochs, overdispersion of 50, and trinucleotide normalization. Each decomposition rank was run with 10 iterations. TensorSignatures was not applied to scenarios 13 and 14 as the estimated computation time, even with multiple GPUs, was expected to be more than 6 months per scenario.

### Quantification and statistical analysis

The quantitative and statistical analyses are described in the relevant sections of the Method details and in the figure legends.

### Additional resources

The novel mutational signatures identified in the present study were included within the reference set of mutational signatures, available at the COSMIC Mutational Signatures website (https://cancer.sanger.ac.uk/signatures/).

## Data Availability

Our study analyzes synthetically generated data, as well as publicly available data from human subjects. The accessions numbers for the human datasets are listed in the key resources table and Table S8, and correspond to a total of 263 published studies as well as 35 ICGC projects.^6^^,^^43^^,^^49^^,^^51^^,^^59^^,^^60^^,^^61^^,^^62^^,^^63^^,^^64^^,^^65^^,^^66^^,^^67^^,^^68^^,^^69^^,^^70^^,^^71^^,^^72^^,^^73^^,^^74^^,^^75^^,^^76^^,^^77^^,^^78^^,^^79^^,^^80^^,^^81^^,^^82^^,^^83^^,^^84^^,^^85^^,^^86^^,^^87^^,^^88^^,^^89^^,^^90^^,^^91^^,^^92^^,^^93^^,^^94^^,^^95^^,^^96^^,^^97^^,^^98^^,^^99^^,^^100^^,^^101^^,^^102^^,^^103^^,^^104^^,^^105^^,^^106^^,^^107^^,^^108^^,^^109^^,^^110^^,^^111^^,^^112^^,^^113^^,^^114^^,^^115^^,^^116^^,^^117^^,^^118^^,^^119^^,^^120^^,^^121^^,^^122^^,^^123^^,^^124^^,^^125^^,^^126^^,^^127^^,^^128^^,^^129^^,^^130^^,^^131^^,^^132^^,^^133^^,^^134^^,^^135^^,^^136^^,^^137^^,^^138^^,^^139^^,^^140^^,^^141^^,^^142^^,^^143^^,^^144^^,^^145^^,^^146^^,^^147^^,^^148^^,^^149^^,^^150^^,^^151^^,^^152^^,^^153^^,^^154^^,^^155^^,^^156^^,^^157^^,^^158^^,^^159^^,^^160^^,^^161^^,^^162^^,^^163^^,^^164^^,^^165^^,^^166^^,^^167^^,^^168^^,^^169^^,^^170^^,^^171^^,^^172^^,^^173^^,^^174^^,^^175^^,^^176^^,^^177^^,^^178^^,^^179^^,^^180^^,^^181^^,^^182^^,^^183^^,^^184^^,^^185^^,^^186^^,^^187^^,^^188^^,^^189^^,^^190^^,^^191^^,^^192^^,^^193^^,^^194^^,^^195^^,^^196^^,^^197^^,^^198^^,^^199^^,^^200^^,^^201^^,^^202^^,^^203^^,^^204^^,^^205^^,^^206^^,^^207^^,^^208^^,^^209^^,^^210^^,^^211^^,^^212^^,^^213^^,^^214^^,^^215^^,^^216^^,^^217^^,^^218^^,^^219^^,^^220^^,^^221^^,^^222^^,^^223^^,^^224^^,^^225^^,^^226^^,^^227^^,^^228^^,^^229^^,^^230^^,^^231^^,^^232^^,^^233^^,^^234^^,^^235^^,^^236^^,^^237^^,^^238^^,^^239^^,^^240^^,^^241^^,^^242^^,^^243^^,^^244^^,^^245^^,^^246^^,^^247^^,^^248^^,^^249^^,^^250^^,^^251^^,^^252^^,^^253^^,^^254^^,^^255^^,^^256^^,^^257^^,^^258^^,^^259^^,^^260^^,^^261^^,^^262^^,^^263^^,^^264^^,^^265^^,^^266^^,^^267^^,^^268^^,^^269^^,^^270^^,^^271^^,^^272^^,^^273^^,^^274^^,^^275^^,^^276^^,^^277^^,^^278^^,^^279^^,^^280^^,^^281^^,^^282^^,^^283^^,^^284^^,^^285^^,^^286^^,^^287^^,^^288^^,^^289^^,^^290^^,^^291^^,^^292^^,^^293^^,^^294^^,^^295^^,^^296^^,^^297^^,^^298^^,^^299^^,^^300^^,^^301^^,^^302^^,^^303^^,^^304^^,^^305^^,^^306^^,^^307^^,^^308^^,^^309^^,^^310^^,^^311^^,^^312^^,^^313^^,^^314^^,^^315^^,^^316^^,^^317^^,^^318^^,^^319^^,^^320^^,^^321^^,^^322^ All results from the benchmarking with synthetic datasets, including the appropriate input used to run each of the tools as well as the output generated by each of the tools, can be found at: ftp://alexandrovlab-ftp.ucsd.edu/pub/publications/Islam_et_al_SigProfilerExtractor/Benchmark/ and Figshare: https://doi.org/10.6084/m9.figshare.20409430. All results from the benchmarking of the different options available in SigProfilerExtractor for matrix normalization, NMF initialization, and NMF objective function can be found at: ftp://alexandrovlab-ftp.ucsd.edu/pub/publications/Islam_et_al_SigProfilerExtractor/Benchmark_Initialization_Normalization_Objective-Function/ and Figshare: https://doi.org/10.6084/m9.figshare.20411483. All results from the *de novo* extraction of mutational signatures from the Pan-Cancer Analysis of Whole Genomes (PCAWG) dataset can be found at: ftp://alexandrovlab-ftp.ucsd.edu/pub/publications/Islam_et_al_SigProfilerExtractor/PCAWG_Reanalysis/ and Figshare: https://doi.org/10.6084/m9.figshare.20406279. All results from the *de novo* extraction of mutational signatures from the extended dataset can be found at: ftp://alexandrovlab-ftp.ucsd.edu/pub/publications/Islam_et_al_SigProfilerExtractor/Extended_Cohort_Reanalysis/ and Figshare: https://doi.org/10.6084/m9.figshare.20406326. A summarized collection of all input mutational matrices, as well as *de novo* extracted mutational signatures and activities for both PCAWG and extended datasets has also been deposited at Figshare: https://doi.org/10.6084/m9.figshare.20293890. All results from the *de novo* extraction of mutational signatures for confirming the patterns of the novel signatures for additional datasets can be found at: ftp://alexandrovlab-ftp.ucsd.edu/pub/publications/Islam_et_al_SigProfilerExtractor/Confirmation_of_Novel_Signatures/ and Figshare: https://doi.org/10.6084/m9.figshare.20406156. All results from the *de novo* extraction of mutational signatures from downsampling of whole-genome sequenced samples to whole-exomes can be found at: ftp://alexandrovlab-ftp.ucsd.edu/pub/publications/Islam_et_al_SigProfilerExtractor/Downsampling_of_whole_genomes/ and Figshare: https://doi.org/10.6084/m9.figshare.20406276. All original code has been deposited at GitHub (https://github.com/AlexandrovLab/SigProfilerExtractor and https://github.com/AlexandrovLab/SigProfilerExtractorR), PyPI (https://pypi.org/project/SigProfilerExtractor/) and Zenodo (https://doi.org/10.5281/zenodo.6746540 and https://doi.org/10.5281/zenodo.6941779). SigProfilerExtractor and all its modules are open source and freely available for use under the permissive 2-clause BSD license. SigProfilerExtractor and its modules are implemented in Python with an R wrapper package allowing users to run the tool from an R environment. The R version of the tool can be downloaded from https://github.com/AlexandrovLab/SigProfilerExtractorR. A detailed wiki page including installation, usage, and explanation of results is provided at https://osf.io/t6j7u/wiki/home/. SigProfilerExtractor is compatible with Windows, Linux, Unix, and macOS operating systems. Any additional information required to reanalyze the data reported in this paper is available from the lead contact upon request.

## Supplemental information

Document S1. Figures S1 and S2

Table S1. Detailed performance metrics after applying each tool across all SBS-96 noiseless synthetic scenarios emulating whole-genome sequencing data, related to Figures 2 and 3A Performance metrics are calculated as per *Figure S1*. An extracted signature is considered a true positive if it matches one of the ground-truth signatures with a cosine similarity threshold of at least 0.90.

Table S2. Detailed performance metrics of applying SigProfilerExtractor and SignatureAnalyzer to extended synthetic scenarios emulating whole-genome sequencing data, related to STAR Methods Performance metrics are calculated as per *Figure S1*. An extracted signature is considered a true positive if it matches one of the ground-truth signatures with a cosine similarity threshold of at least 0.90.

Table S3. Detailed performance metrics after applying the eight best-performing tools across SBS-96 synthetic scenarios emulating whole-genome sequencing data with different levels of noise, related to Figure 3B and 3C Performance metrics are calculated as per *Figure S1*. An extracted signature is considered a true positive if it matches one of the ground-truth signatures with a cosine similarity threshold of at least 0.90.

Table S4. Shannon equitability index for false positive signatures extracted by the eight best-performing tools across SBS-96 synthetic scenarios emulating whole-genome sequencing data with different levels of noise, related to STAR Methods The Shannon equitability index is calculated as described in the *STAR Methods*. Signatures showing flat, featureless mutational profiles show equitability indexes near one, while sparse signatures display values closer to zero.

Table S5. Detailed performance metrics after applying the eight best-performing tools across SBS-96 synthetic scenarios emulating whole-exome sequencing data with 5% noise, related to Figure 3D Performance metrics are calculated as per *Figure S1*. An extracted signature is considered a true positive if it matches one of the ground-truth signatures with a cosine similarity threshold of at least 0.90.

Table S6. Detailed performance metrics of applying the different options available in SigProfilerExtractor for matrix normalization, NMF initialization, and NMF objective function based on SBS-96 synthetic scenarios emulating whole-genome sequencing data with 5% noise, related to STAR Methods and Figure S2 Performance metrics are calculated as per *Figure S1*. An extracted signature is considered a true positive if it matches one of the ground-truth signatures with a cosine similarity threshold of at least 0.90.

Table S7. Profiles of four novel mutational signatures identified in a cohort of 4,643 whole-genome-sequenced and 19,184 whole-exome-sequenced human cancers, related to Figure 4 The profiles of the novel mutational signatures are reported using the SBS-288 classification which incorporates the trinucleotide context and strand information (intergenic region, untranscribed strand, or transcribed strand) for each type of single base substitution. The SBS-288 classification can be easily collapsed to the commonly used SBS-96 classification.

Table S8. Sources of somatic mutation data for 4,643 whole-genome-sequenced and 19,184 whole-exome-sequenced human cancers, related to STAR Methods Project codes and/or digital object identifiers (DOI) and PubMed IDs (PMID) for the original publications are listed for every cohort.

Table S9. Ranges and ground-truth numbers of mutational signatures used for the 34 synthetic scenarios, related to STAR Methods Ranges of signatures used for the suggested model selection and ground-truth numbers of signatures used for the forced model selection. The same range used for noiseless scenarios 11 and 12 was applied for the WGS noise scenario. A reduced range was used for WES noise scenario to optimize running time, except for SigneR, whose signature selection method depends on the maximum number of signatures tested (original WGS range was applied).

Document S2. Article plus supplemental information
