# Supplementary material for: Uncovering novel mutational signatures by de novo extraction with SigProfilerExtractor
Source: Cell Genom. 2022 Sep 23;2(11):100179. doi: 10.1016/j.xgen.2022.100179 (PMC9646490; doi:10.1016/j.xgen.2022.100179)

Cell Genomics, Volume *2*

Supplemental information

Uncovering novel mutational signatures

by *de novo* extraction with SigProfilerExtractor

S.M. Ashiqul Islam, Marcos Díaz-Gay, Yang Wu, Mark Barnes, Raviteja Vangara, Erik N. Bergstrom, Yudou He, Mike Vella, Jingwei Wang, Jon W. Teague, Peter Clapham, Sarah Moody, Sergey Senkin, Yun Rose Li, Laura Riva, Tongwu Zhang, Andreas J. Gruber, Christopher D. Steele, Burçak Otlu, Azhar Khandekar, Ammal Abbasi, Laura Humphreys, Natalia Syulyukina, Samuel W. Brady, Boian S. Alexandrov, Nischalan Pillay, Jinghui Zhang, David J. Adams, Iñigo Martincorena, David C. Wedge, Maria Teresa Landi, Paul Brennan, Michael R. Stratton, Steven G. Rozen, and Ludmil B. Alexandrov

Supplementary Figures

Figure S1. Standard set of performance metrics used for benchmarking all bioinformatics tools, Related to Figures 2 and 3. An example demonstrating the derivation of *true positive* (TP), *false positive* (FP), or *false negative* (FN) signatures for a tool applied to a synthetic dataset generated using 6 ground-truth signatures (termed, Ground-Truth Signatures 1 through 6). The tool extracts 4 signatures (termed, Extracted Signatures A through D). In this example, an extracted signature is considered a true positive if it matches one of the ground-truth signatures with a cosine similarity threshold of at least 0.90.

Simulated *dataset* using 6 ground truth signatures (Ground Truth Signatures 1 through 6). A tool extracts 4 signatures (Extracted Signatures A through D). Comparison between Ground Truth and Extracted Signatures using cosine similarity.

	Extracted Signature A	Extracted Signature B	Extracted Signature C	Extracted Signature D
Ground Truth Signature 1	0.14	0.98	0.56	0.36
Ground Truth Signature 2	0.35	0.29	0.93	0.46
Ground Truth Signature 3	0.31	0.56	0.78	0.66
Ground Truth Signature 4	0.34	0.08	0.57	0.67
Ground Truth Signature 5	0.95	0.15	0.81	0.39
Ground Truth Signature 6	0.23	0.74	0.48	0.26

True Positives (TP; ≥ 0.90)

Extracted Signature A
Extracted Signature B
Extracted Signature C

Signatures correctly extracted from the *dataset*

False Positives (FP)

Extracted Signature D

Signatures extracted but absent in the *dataset*

False Negatives (FN)

Ground Truth Signature 3
Ground Truth Signature 4
Ground Truth Signature 6

Signatures not extracted but used in simulating the *dataset*

Cosine similarity between Extracted Signature C and Ground Truth Signature 6

Figure S2. Comparison of the different options available in SigProfilerExtractor for matrix normalization, NMF initialization, and NMF objective function, Related to STAR Methods. Vertical axes reflect F_1 score (left plot), sensitivity (middle plot), and false discovery rate (right plot), respectively. Abbreviations: gmm: Gaussian mixture model; nndsvd_min: nonnegative double singular vector decomposition initialization where zeros are replaced by the minimum positive value.

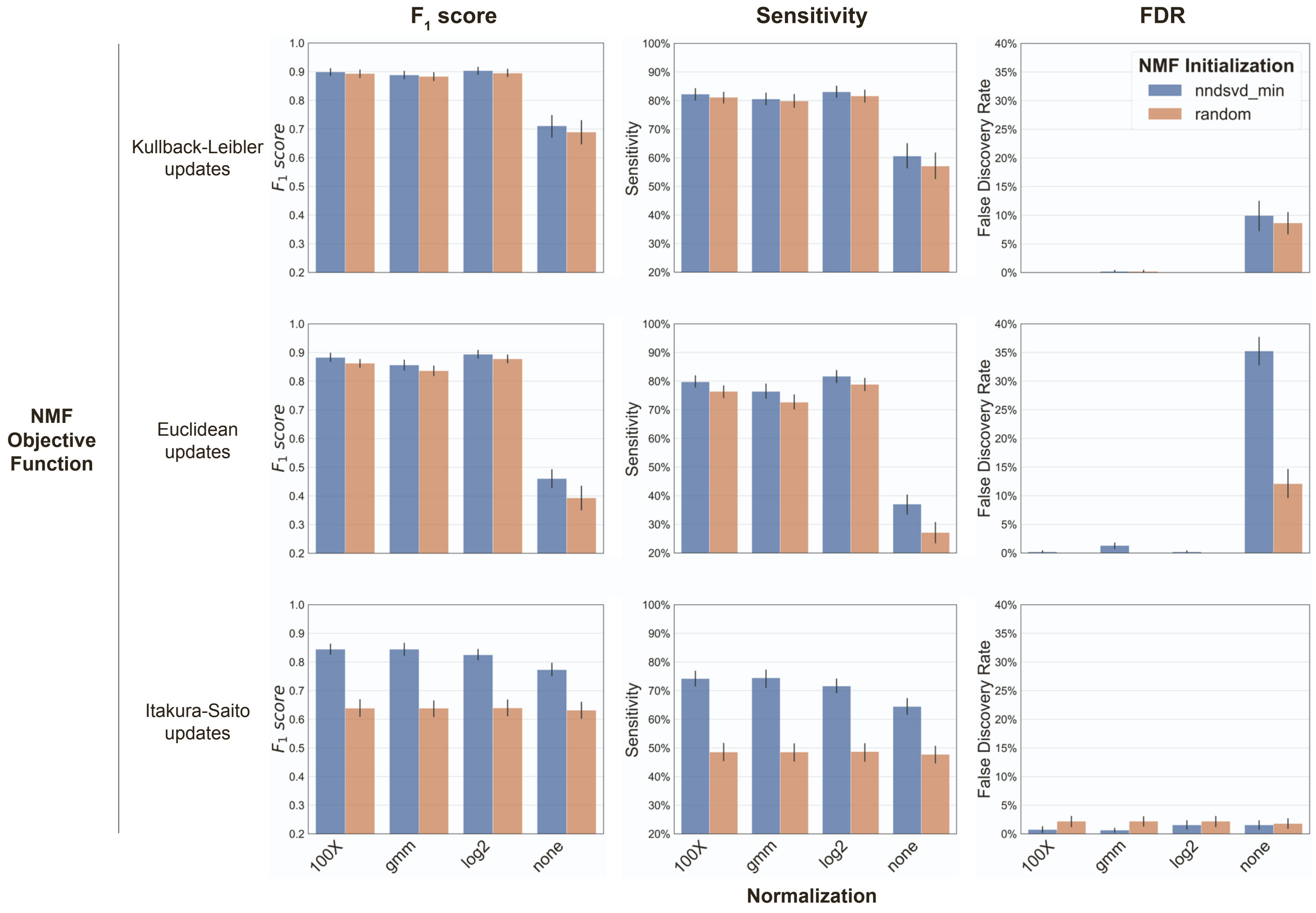

Supplement: Document S1. Figures S1 and S2 [file mmc1.pdf]
